# Testing for shared biogeographic history in the lower Central American freshwater fish assemblage using comparative phylogeography: concerted, independent, or multiple evolutionary responses?

**DOI:** 10.1002/ece3.1058

**Published:** 2014-04-10

**Authors:** Justin C Bagley, Jerald B Johnson

**Affiliations:** 1 Evolutionary Ecology Laboratories, Department of Biology, Brigham Young UniversityProvo, Utah, 84602; 2 Monte L. Bean Life Science Museum, Brigham Young UniversityProvo, Utah, 84602

**Keywords:** Comparative phylogeography, freshwater fishes, hierarchical approximate Bayesian computation, Neotropics, Nicaraguan depression, Poeciliidae

## Abstract

A central goal of comparative phylogeography is determining whether codistributed species experienced (1) concerted evolutionary responses to past geological and climatic events, indicated by congruent spatial and temporal patterns (“concerted-response hypothesis”); (2) independent responses, indicated by spatial incongruence (“independent-response hypothesis”); or (3) multiple responses (“multiple-response hypothesis”), indicated by spatial congruence but temporal incongruence (“pseudocongruence”) or spatial and temporal incongruence (“pseudoincongruence”). We tested these competing hypotheses using DNA sequence data from three livebearing fish species codistributed in the Nicaraguan depression of Central America (*Alfaro cultratus*, *Poecilia gillii*, and *Xenophallus umbratilis*) that we predicted might display congruent responses due to co-occurrence in identical freshwater drainages. Spatial analyses recovered different subdivisions of genetic structure for each species, despite shared finer-scale breaks in northwestern Costa Rica (also supported by phylogenetic results). Isolation-with-migration models estimated incongruent timelines of among-region divergences, with *A. cultratus* and *Xenophallus* populations diverging over Miocene–mid-Pleistocene while *P. gillii* populations diverged over mid-late Pleistocene. Approximate Bayesian computation also lent substantial support to multiple discrete divergences over a model of simultaneous divergence across shared spatial breaks (e.g., Bayes factor [*B*_10_] = 4.303 for *Ψ* [no. of divergences] > 1 vs. *Ψ* = 1). Thus, the data support phylogeographic pseudoincongruence consistent with the multiple-response hypothesis. Model comparisons also indicated incongruence in historical demography, for example, support for intraspecific late Pleistocene population growth was unique to *P. gillii*, despite evidence for finer-scale population expansions in the other taxa. Empirical tests for phylogeographic congruence indicate that multiple evolutionary responses to historical events have shaped the population structure of freshwater species codistributed within the complex landscapes in/around the Nicaraguan depression. Recent community assembly through different routes (i.e., different past distributions or colonization routes), and intrinsic ecological differences among species, has likely contributed to the unique phylogeographical patterns displayed by these Neotropical fishes.

## Introduction

Comparative phylogeographic studies provide an important means of elucidating the relative influence of shared earth history events on contemporary biodiversity. By comparing spatial-genetic divergences, divergence times, gene flow, and population dynamics (e.g., *N*_e_, effective population size) across multiple codistributed species, comparative studies provide critical assessments of phylogeographical congruence, forming a basis for historical inferences (Bermingham and Martin 1998; Avise 2000; Arbogast and Kenagy 2001; Hickerson et al. 2010). Any of several outcomes may result, embodied by at least three general competing hypotheses with unique biogeographical implications. The first hypothesis, the “concerted-response hypothesis” predicts that codistributed species responded in lockstep fashion to geological and palaeoclimatic events, and correlated habitat shifts, within their overlapping distributions (Sullivan et al. 2000). Concerted responses are supported by congruent genetic breaks across taxa in space and time (Donoghue and Moore 2003), which should be common among ecologically and phylogenetically similar taxa due to codependence on similar habitats (Bermingham and Martin 1998; Feldman and Spicer 2006). Congruent responses point to causal factors underlying diversification and are consistent with long coassociations in local communities resulting in similar evolutionary trajectories (Avise 2000). Comparative phylogeographic congruence also predicts similar patterns in codistributed yet un-sampled taxa (Avise 2000; Sullivan et al. 2000).

One alternative to the concerted-response hypothesis, the “independent-response hypothesis”, predicts codistributed species will bear genetic signatures of independent evolutionary responses to regional historical processes (Sullivan et al. 2000). This hypothesis is supported by phylogeographical incongruence in space, not time. This is because “incongruence” is identified when different spatial-genetic breaks derive from synchronous diversification (Cunningham and Collins 1994; Donoghue and Moore 2003). Incongruence thus occurs because species show different responses to the same earth history events or to the same deterministic biological factors (e.g., predation environment), and such independent but synchronous evolutionary trajectories are thought most likely to arise due to intrinsic differences in biological attributes among the species sampled (Cunningham and Collins 1994; Bermingham and Martin 1998; Avise 2000; Arbogast and Kenagy 2001; Donoghue and Moore 2003).

In contrast with the two competing hypotheses above, which propose temporal congruence, “pseudocongruence” and “pseudoincongruence” arise when spatial-genetic divergences are respectively congruent or incongruent but asynchronous, reflecting different responses correlated to different events (Cunningham and Collins 1994; Donoghue and Moore 2003). We refer to these scenarios defined by temporal incongruence as variations of a “multiple-response hypothesis”. Complex pseudocongruent or pseudoincongruent patterns indicate little or no history of community coevolution; rather, different past distributions and recent community assembly, or stochastic dispersal or lineage sorting events, best explain such phylogeographic patterns (e.g., Donoghue and Moore 2003; Nielsen and Beaumont 2009). Biological factors also influence multiple-response scenarios, for example, to the extent that ecological differences determine species propensities or rates of dispersing into and becoming established in novel areas.

The lower Central American (LCA) isthmus is famous worldwide as an example of a land-bridge formation that has shaped continental Neotropical biotas by facilitating widespread dispersals, speciation, and extinctions in North and South America (Marshall et al. 1979; Stehli and Webb 1985). However, the LCA subcontinent is also increasingly appreciated as a system of highly endemic assemblages with interwoven histories of community assembly and species diversification (Bermingham and Martin 1998; Reeves and Bermingham 2006; Bagley and Johnson 2014). Much of our understanding of this history comes from studies of LCA species phylogeographic histories conducted in recent years (reviewed by Bagley and Johnson 2014). The LCA freshwater fish assemblage presents a particularly interesting model for phylogeography. This group is composed of >170 species from ecologically and morphologically diverse clades, including a wide representation of the teleost families Cichlidae (33 species) and Poeciliidae, that is, “livebearing fishes” (∼28 species) (Bussing 1998; Smith and Bermingham 2005). Many species in this assemblage occupy identical river systems, making them well suited to test for concerted evolutionary responses, as they were potentially affected by the same past environmental changes (Bermingham and Martin 1998; Smith and Bermingham 2005). Previous studies indicate that freshwater fishes colonized LCA in multiple waves, mostly during the Pliocene–Pleistocene, but were more restricted than terrestrial taxa in tracking habitat disturbances as orogeny and drainage boundary formation progressed (Bermingham and Martin 1998; Streicher et al. 2009; Loaiza et al. 2010). This led to cryptic dispersals, vicariance, drainage isolation, and speciation within the assemblage (e.g., Martin and Bermingham 2000). For example, mtDNA phylogeography studies have recovered complex, but spatially correlated, genetic breaks across multiple fish species in Panama and between LCA and northwestern South America (e.g., Bermingham and Martin 1998; Martin and Bermingham 2000; Perdices et al. 2005; Reeves and Bermingham 2006), suggesting potential commonalities of evolutionary history (reviewed by Bagley and Johnson 2014). However, a limited subset of species phylogeographies have been inferred to date, compared with the total species diversity of the LCA fish assemblage. Thus, the question of whether LCA freshwater fish communities experienced common spatial, temporal, and demographic responses to past environmental changes, or not, remains an open one, particularly for northern LCA (Costa Rica), where few phylogeography studies to date have sampled freshwater fishes (Bagley and Johnson 2014).

**Figure 1:**
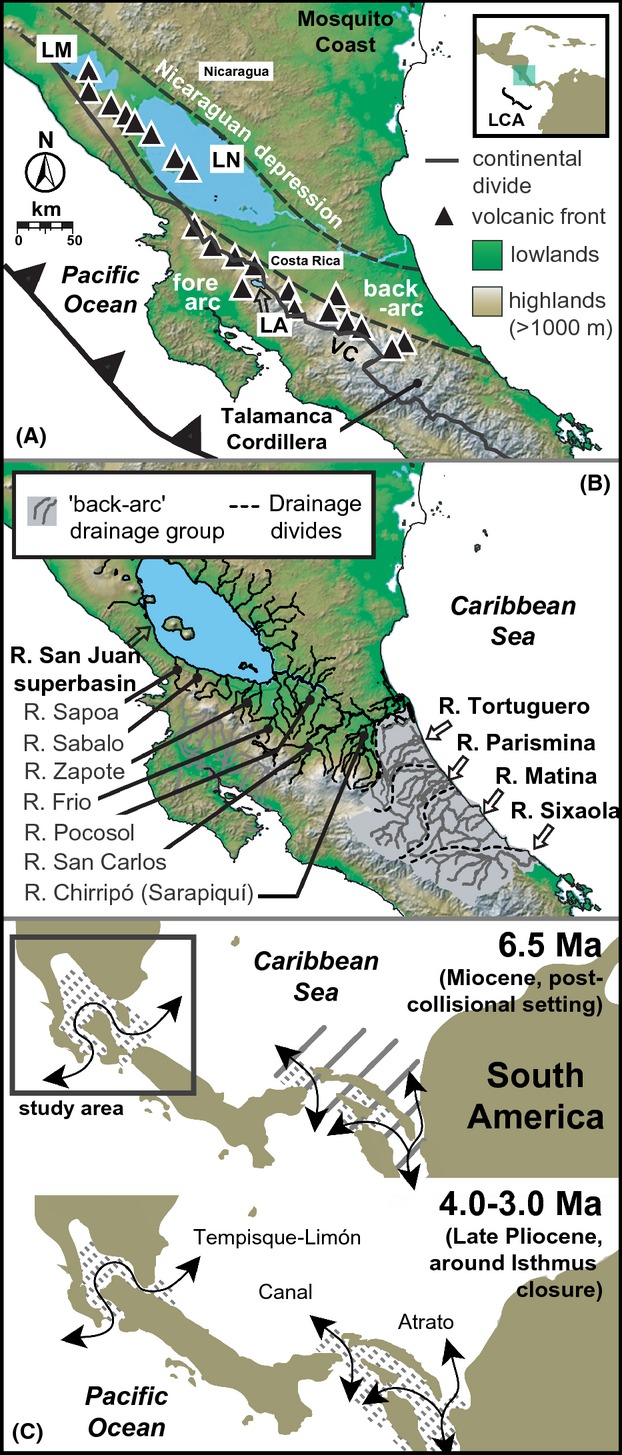
Study area. Major physiographic elements include: (A) the Nicaraguan depression (ND), Lakes Managua (LM), and Nicaragua (LN), the Rio San Juan superbasin (SJ), Lake Arenal (LA), the Valle Central (VC), and Quaternary stratovolcanoes (black triangles with white trim); inset map: position relative to greater Central American/Caribbean realm. Drainages subdivide into two a priori drainage groups (B): tributaries connected through freshwater in the San Juan superbasin (dark lines), and Caribbean “back-arc” drainages (light gray shading, dotted black borders). Palaeogeographic reconstructions (C; after Coates and Obando 1996; Coates et al. 2004) indicate that the study area was partly inundated by marine corridors (arrows) over Miocene–Pliocene (brown, land; diagonal lines, abyssal depths; stippling, neritic depths). Digital elevation layers were derived from NASA Shuttle Radar Topography Mission image PIA03364.

Here, we conduct a comparative analysis of three species from the LCA freshwater fish assemblage, in order to empirically evaluate predictions of the concerted-, independent-, and multiple-response hypotheses. Within LCA, we focus on the Nicaraguan depression (ND; Fig. 1A) and surrounding uplands of the San Juan biogeographical province (Bussing 1985, 1998; Smith and Bermingham 2005), where a unique history of factors likely influenced species evolution (discussed below) and several livebearing fish species ranges overlap (Bussing 1998). Phylogeography studies have been conducted on two livebearers from this area: *X. umbratilis* (Meek 1912) (monotypic, hereafter “*Xenophallus*”), and molly, *P. gillii* (Kner 1863). Within *Xenophallus*, Jones and Johnson (2009) discovered two deeply-diverged mitochondrial (mt) DNA lineages in the upland San Carlos basin, and the ND lowlands (Fig. 1) presumably correlated with sea-level dynamics; genetic diversification since the Pliocene (∼4.5 Ma); and significant genetic partitioning by drainages. In contrast, Lee and Johnson (2009) found evidence for shallow haplotype divergences, limited among-region differentiation, and complex gene flow between populations of *P. gillii* from the same area. However, Bayesian demographic models published by Jones and Johnson (2009) and Lee and Johnson (2009) seemingly indicate overlapping Pleistocene-recent population bottleneck events in these taxa, assuming similar mtDNA substitution rates. These studies denote progress toward comparative perspectives on the evolutionary history of northern LCA freshwater communities. However, these species phylogeographies have never been rigorously compared using identical analyses. Thus, their degree of spatial and temporal congruence, and whether their genetic patterns represent general evolutionary patterns, remains unclear. By combining phylogeographical analyses of new DNA sequences from knife-edged livebearer, *A. cultratus* (Regan 1908), with analyses of existing data from *P. gillii* and *Xenophallus* populations codistributed in the ND, we test two predictions of the concerted-response hypothesis, against the independent- and multiple-response hypotheses. These predictions include (1) that these fishes should exhibit congruent spatial-genetic structuring and (2) that spatial subdivisions should be temporally congruent, due to co-occurrence in drainages correlated with LCA geomorphology (Marshall et al. 2003; Smith and Bermingham 2005). Additionally, coalescent theory predicts that genetic variation changes with historical *N*_e_, for example, during population growth, decline, or range shifts (Wakeley 2000, 2003). Thus, to enhance our understanding of potential connections between historical biogeography and demography in these species, we also evaluate intraspecific DNA polymorphism and neutrality, and then compare historical-demographic responses among species.

## Materials and Methods

### Study area and sampling

The study area encompasses ∼18,000 km^2^ in and around the ND in southern Nicaragua and northern Costa Rica (Fig. 1). Here, our focal species co-occur in five major drainages at elevations ranging from 35 to 346 m. Based on geomorphology, these drainages subdivide into two a priori groups shown in Figure 1B: (1) the Rio San Juan superbasin, including lakes Managua and Nicaragua and tributaries to the southeast associated with Pliocene–Holocene formations of the Chorotega volcanic front (Fig. 1A); and (2) four Caribbean drainages along the LCA “back-arc” isolated from each other by saltwater, whose headwaters are associated with the Miocene–recent Talamanca Cordillera (Marshall 2007). The ND is a long, fault-bounded rift valley spanning El Salvador's Median Trough to the Tortuguero lowlands basin, Costa Rica. The ND formed by extensional forces at LCA's northern boundary, resulting in southeast–northwestward opening since 10 Ma (Mann et al. 2007; Funk et al. 2009). Sedimentary records show that, during Miocene–Pliocene high seas ∼50–100 m above present sea level and even moderate late-Pliocene seas (Haq et al. 1987; Miller et al. 2005), a marine corridor inundated the ND until at least late Pliocene (Fig. 1C; Coates and Obando 1996; Coates et al. 2004). This corridor limited dispersal of many organisms between Nicaragua and Costa Rica, including freshwater fishes (Bussing 1976). Subsequently, the ND study area was above water (had surface freshwaters) by ∼3.0–2.1 Ma (Coates and Obando 1996; Marshall et al. 2003). Proto-Chirripó drainage headwaters were redirected to the Pacific during creation of the Valle Central ∼0.8–0.3 Ma (Fig. 1A; Marshall et al. 2003). The nearby Central Cordillera formed by Late Pleistocene, leaving active volcanoes amid drainage headwaters (Fig. 1A; Marshall et al. 2003), a source of periodic local extinctions. Because the steep Caribbean continental shelf restricts river anastomosis, over 100 m drops in sea level during Pleistocene glacial maxima (Lambeck et al. 2002) probably altered coastal freshwater connectivity minimally in this area (Smith and Bermingham 2005). Thus drainages modulated in length and elevation during Pleistocene sea-level cycles associated with glacial stages. Whereas Pleistocene–recent patterns of sea-level fall are widely agreed upon, the extent of eustatic sea-level highstands of the Quaternary remains debated among geologists; however, available data indicate large correlated spikes ≥20–30 m above present sea level ∼2.4–1.8 Ma and 1.3 Ma (Miller et al. 2005) and ∼550 ka (Hearty et al. 1999), that probably inundated LCA lowlands. Each of these Pliocene–Holocene environmental disturbances might have importantly shaped ND species phylogeographies, producing range fragmentation, upland isolation, or extinction-recolonization dynamics.

**Figure 2:**
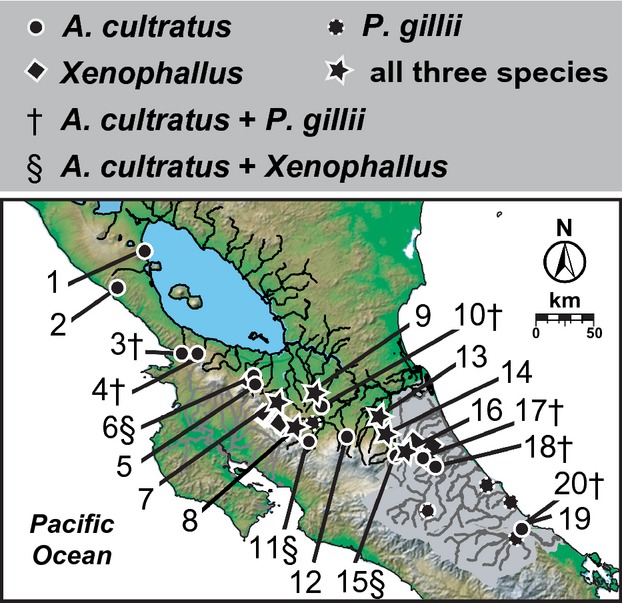
Geographical sampling localities. Sites where *Alfaro cultratus* (circles, 1–20), *Poecilia gillii* (dotted circles), and *Xenophallus umbratilis* (diamonds) were sampled are shown, including sites where we sampled all three species (stars) and combinations of two species (indicated by † and § symbols next to site numbers). Sites correspond to exact localities and sample sizes listed in Table S1.

We sampled *A. cultratus* from 18 localities (sites 3–20 in Fig. 2) in four of the five study area drainages (Fig. 1B). We obtained samples of *Alfaro huberi* (Fowler 1923), the allopatric sister species to *A. cultratus*, from five sites in Honduras. Specimens were preserved in 95% ethanol in the field. We augmented data from these samples with published mtDNA cytochrome *b* (cyt*b*) sequences from *A. cultratus* [*N* = 3, sites 1–2; from Hrbek et al. (2007), Doadrio et al. (2009)] and codistributed *P. gillii* and *Xenophallus* populations (Jones and Johnson 2009; Lee and Johnson 2009), including 19 *P. gillii* localities and 23 *Xenophallus* localities (Fig. 2). There were six sites in the San Juan and Tortuguero drainages where we sampled all three species. Table S1 provides detailed sampling data and GenBank accession numbers. Outgroups used in the analyses below are described in Appendix S1.

### Laboratory methods

We collected DNA sequence data from *A. cultratus* and *A. huberi* samples. After isolating DNA using the Qiagen DNeasy96 tissue protocol (Qiagen Sciences, Germantown, MD, USA), we amplified cyt*b* fragments for each sample by PCR using forward primer GLU31 (Unmack et al. 2009) and reverse primer HD (15680; Schmidt et al. 1998). Amplification and sequencing reactions, clean up, and sequence visualization followed Lee and Johnson (2009). We aligned mtDNA sequences manually in SEQUENCHER 4.8 (Gene Codes Corporation, Ann Arbor, MI, USA) and checked amino acid coding for errors (stop codons) while viewing electropherograms. We collapsed identical cyt*b* sequences into unique haplotypes using DnaSP 5.10 (Librado and Rozas 2009). We obtained a total of 355 *A. cultratus* and seven *A. huberi* sequences of a cyt*b* fragment 601 bp in length. Cyt*b* data encompassed 46 *A. cultratus* haplotypes, 37 *P. gillii* haplotypes (from 143 sequences; 1140 bp), and 29 *Xenophallus* haplotypes (from 131 sequences; 1140 bp), plus additional outgroup sequences.

Analyzing multiple unlinked loci can improve phylogeographical inferences, including population divergence-time and summary-statistics estimates (Edwards and Beerli 2000; Wakeley 2003), and provide perspective on putative sex-based asymmetries in gene flow and population structure (e.g., Avise 2000; Zink and Barrowclough 2008). Thus, we additionally screened nuclear ribosomal protein *S7* (*RPS7*; *N* = 72) introns 1 and 2 from multiple *A. cultratus* populations. Unfortunately, these sequences were uninformative in pilot analyses (e.g., star phylogeny, ∼0.8% overall pairwise divergence), so we excluded them from our analyses. One limitation of basing our phylogeographical inferences on the matrilineal signal of mitochondrial DNA is that our results may not necessarily be congruent with patterns of population history in nuclear genomes. Despite such concerns, we are confident that our mtDNA analyses are appropriate for the questions we have addressed; for example, mtDNA is a robust indicator of population history and species histories, especially across multiple codistributed taxa, and thus has been a workhorse of comparative phylogeography (e.g., due to high information content, faster coalescence, etc., Avise 2000; Zink and Barrowclough 2008). Moreover, our use of mitochondrial markers makes our results comparable to several other LCA studies (e.g., Sullivan et al. 2000; Jones and Johnson 2009; Lee and Johnson 2009).

### Genetic diversity and neutrality

**Figure 3:**
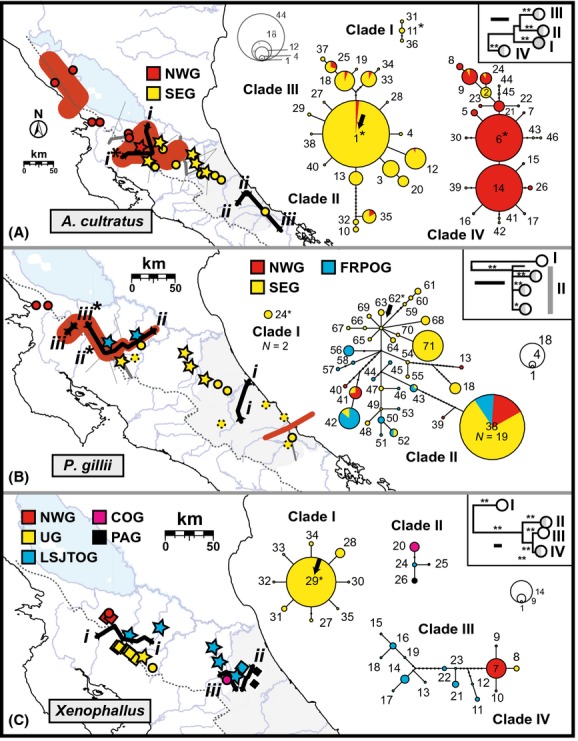
Incongruent spatial-genetic structuring among Nicaraguan depression livebearing fish species, based on mtDNA cyt*b* variation. Solid black lines indicate genetic barriers (i–iii) delimiting distinct population groups (represented with different colors and abbreviations; population groups are described in the text) inferred using Monmonier's algorithm in BARRIER. Asterisks indicate significant barriers, based on bootstrapping; and maximum TrN genetic distances across each break are given as percentages. Table 1 presents summary-statistics and neutrality tests for these groups. Corresponding cyt*b* parsimony networks are also shown presenting haplotypes as network circles, scaled according to their frequency and colored to show proportions of their distributions in each population group. Networks were separated based on a 95% parsimony criterion. Phylogenetic relationships are shown within each species map (inset boxes), with nodal support (BP: *50–70, **>70) and a scale bar (0.01 subs/site) for the simplified maximum-likelihood tree; tip circles summarize clade geography with respect to drainage groups in Figure 1B (dark gray, San Juan drainage; light gray, back-arc). Haplotype numbers correspond to labels used in (A) Table S1, (B) Lee and Johnson (2009), and (C) Jones and Johnson (2009).

We compared intraspecific genetic diversity levels across taxa by calculating segregating sites (*S*), haplotype diversity (*Hd* ± SE [standard error]), nucleotide diversity (*π*), and Watterson's (1975) *θ*_w_ (per site) for each locality and species using DnaSP. We calculated the same summary statistics in DnaSP for each population group (see BARRIER Results, Fig. 3). We also computed summary-statistic averages across localities within drainages. Patterns captured by these statistics may reflect sampling differences, for example, denser within-locality sampling in *A. cultratus*; however, *Hd* and *π* are less sensitive to such sampling effects (Li 1997). We assessed selective neutrality of each cyt*b* dataset–an assumption of most of our analyses–using Hudson-Kreitman-Aguadé (HKA; Hudson et al. 1987) tests, testing significance using 10^4^ coalescent simulations in DnaSP; these tests used outgroups identical to phylogenetic outgroups below.

### Spatial patterns

To test for spatial-genetic congruence, as predicted under the concerted-response hypothesis, we evaluated genetic structuring and breaks across the study area while taking spatial sampling patterns into account, but without prior knowledge of population structure or genetic barriers. First, we used the simulated annealing algorithm implemented in SAMOVA 1.0 (Dupanloup et al. 2002) to define genetically homogeneous, maximally differentiated spatial population clusters (*K*). We modeled *K =* 2–10 groups, drawing from 100 initial conditions, and noted fixation index (Φ_*CT*_) trends. Second, we identified genetic barriers among populations using BARRIER 2.2 (Manni et al. 2004a,b). In BARRIER, we laid Delaunay triangulation networks over sampling sites (based on Voroni tessellation). We then used Monmonier's (1973) algorithm to sequentially identify genetic “barriers” as locations of maximum pairwise Tamura and Nei (Tamura and Nei 1993; TrN) genetic distances between localities across each network, calculated in ARLEQUIN 3.5 (Excoffier and Lischer 2010; 1000 nonparametric permutations). We assessed relative support for barriers by calculating bootstrap proportions (BP) from 100 bootstrapped barriers, generated by supplying BARRIER with bootstrapped TrN distance matrices (resampling the original datasets within populations, using PopTools; Hood 2008); we considered it strong support when BP ≥ 50. We did not apply this procedure to *Xenophallus*, because low within-site genetic diversity rendered bootstrapping ineffective. We independently tested spatial configurations inferred in SAMOVA and BARRIER using analyses of molecular variance (AMOVA) performed in ARLEQUIN (1000 nonparametric permutations). When faced with isolation-by-distance, SAMOVA and Monmonier's algorithm are more likely to misidentify populations and genetic barriers between them (Dupanloup et al. 2002). Thus, we tested correspondence between linearized genetic distance [*F*_ST_/(1–*F*_ST_)] and natural log-transformed geographic distance between localities using standard regression, and Mantel tests (Mantel 1967) with significance tested using 10^4^ permutations in PASSAGE 2 (Rosenberg and Anderson 2011). Details of SAMOVA and BARRIER analyses and interpretation are given in Appendix S2.

We also tested for congruent hierarchical genetic partitioning among San Juan basin tributaries and drainage groups using two a priori biogeographic AMOVAs. These AMOVAs were similar to those employed by Jones and Johnson (2009), except we grouped localities using drainage and drainage groups as defined in Figure 1B. We qualitatively tested for similar population groups (SAMOVA, BARRIER), genetic barriers between major drainages (BARRIER, AMOVAS), and among-drainage partitioning across taxa (AMOVAS) to identify shared effects of drainage boundaries as historical barriers to gene flow.

We compared phylogenetic relationships (haplotype gene trees) and nodal support among cyt*b* haplotypes inferred for each focal species using maximum likelihood tree searches and bootstrap (500 pseudoreplicates) searches in GARLI 0.97 (Zwickl 2006). Likelihood analyses relied on substitution models (Table S2) selected using a decision theory algorithm, DT-ModSel (Minin et al. 2003), and partitioned data by codon position, ([1 + 2], 3). We independently inferred relationships among phylogenetic clades using statistical parsimony analyses in TCS 1.21 (Clement et al. 2000; 95% Connection Limit). We estimated sequence divergence over haplotype pairs among clades as pairwise maximum composite likelihood means in MEGA5 (Tamura et al. 2011).

### Temporal patterns

We evaluated temporal congruence, the second prediction of the concerted-response hypothesis, by using the Bayesian coalescent dating approach implemented in IMa2 (Hey 2010) to estimate divergence times (*t*) among adjacent population groups from BARRIER. While we were mainly interested in estimating *t*, IMa2 also estimates population migration rates (*m*_1_, *m*_2_) and sizes of current (*θ*_1_, *θ*_2_) and ancestral populations (*θ*_A_) using Hey and Nielsen's (2004) “isolation-with-migration” model. We conducted several pilot runs to estimate appropriate Markov chain Monte Carlo (MCMC) sampling chain lengths and priors. Subsequently, we ran three final runs per population pair starting from different random seeds, with 10 chains each. After logging 10^6^ states discarded as “burn-in”, we ensured chain mixing and convergence, judged by (1) ≥10% update rates for *t*, (2) appropriate chain-swapping rates, and (3) runs converging on similar parameter estimates. Fossil data and species-specific substitution rates were unavailable to us, thus we specified uniform mutation rate (*μ*) priors spanning lower and upper per-lineage mutation rates published for teleost fish protein-coding mtDNA, 1.7 × 10^−9^ and 1.4 × 10^−8^ substitutions/site/year (subs/site/year) [refs. in Waters and Burridge (1999), Burridge et al. (2008)]. See Appendix S3 for details of our IMa2 runs, for example, prior settings. Resulting *t* estimates were converted to absolute time (*T*_div_) using the equation *T*_div_ = *t*/*μk* (where *k =* sequence length), assuming species generation times equivalent to 1 year/generation (Winemiller 1993). To cover a range of possible mutation rates, we estimated *T*_div_ twice per population pair, setting *μ* equal to (1) the standard 2% rate (1.0 × 10^−8^ subs/site/year, per lineage) for vertebrate mtDNA (Brown et al. 1979; Wilson et al. 1985) and (2) a “slower” 0.9% pairwise rate (4.5 × 10^−9^ subs/site/year, per lineage) estimated for trout species mtDNA (Salmonidae; Martin and Palumbi 1993) that has previously been applied to higher-order teleosts (e.g., Waters and Burridge 1999).

We further tested the temporal congruence of shared genealogical breaks within northwestern Costa Rica (see Results) using hierarchical approximate Bayesian computation as implemented in the bioinformatics pipeline, MTML-msBayes (Huang et al. 2011). Using MTML-msBayes, we tested for simultaneous divergence among three population-group pairs diverged in this area under a finite sites coalescent model, allowing lineages to diverge, experience different migration patterns, and change population sizes (*θ*) independently while accounting for coalescent gene-tree stochasticity (Huang et al. 2011; refs. therein). After calculating a vector of observed summary statistics for each population pair, we used coalescent simulations to generate 5 × 10^6^ simulated DNA datasets for three population pairs. Simulations assumed no migration or recombination, consistent with general IMa2 and neutrality test results (see Results). We generated hyper-posteriors for the mtDNA, representing 1000 random draws from the joint posterior distribution, by comparing the observed versus simulated summary statistics vectors using the pipeline's standard rejection/acceptance algorithm. Posterior estimates of the number of discrete co-divergences (*Ψ*) were obtained via polychotomous regression; posterior estimates of population divergence time (*E*[*τ*]; units of *μ*/generation) and *Ω* (dispersion index representing the ratio of variance to the mean divergence times across *Y* taxon pairs) were obtained by local linear regression (Beaumont et al. 2002). To evaluate the “weight of evidence” in favor of simultaneous divergence, we calculated Bayes factors (*B*_10_) to compare the level of posterior support for simultaneous versus nonsimultaneous divergence (*Ψ* = 1 vs. *Ψ* > 1; and *Ω* = 0.05 vs. *Ω* > 0.05), using Jeffreys' (1961) criteria for *B*_10_ “weight of evidence”. We also used *B*_10_ values to evaluate support for continuous divergence (*Ψ* = 3 vs. *Ψ* < 3). Bayes factor calculations accounted for prior support for each hypothesis. To explore our data, we conducted multiple msBayes runs across a range of prior values (upper *θ* = 0.005–0.05; upper ancestral *θ* = 0.25–0.5; *Nm* = 0) to evaluate the effects of the prior on the models, and we conducted Bayes factor hypotheses testing using each model.

### Historical demographic patterns

We qualitatively evaluated historical demographic congruence by comparing estimates of past population size fluctuations through time captured using the Bayesian skyline plot method (Drummond et al. 2005) implemented in BEAST 1.7.5 (Drummond et al. 2012). Pilot runs (MCMC=10^6^) showed that marginal relaxed clock standard deviations (“ucld.stdev” parameter) clumped at zero, indicating highly clock-like data. Therefore, we conducted Bayesian skyline model runs using strict clocks (MCMC=2 × 10^8^; burn-in=2 × 10^7^; “Piecewise-constant” skyline model; “Coalescent: Constant Size” tree priors). We specified uniform priors spanning teleost mtDNA mutation rates (see IMa2 methods) and substitution models selected by DT-ModSel (Table S2). We partitioned sites by codon position ([1 + 2], 3), unlinking parameters across subsets. We calculated posterior distributions of *N*_e_*τ* through time, and node ages (*t*_MRCA_s), and their 95% highest posterior densities (HPD) using TRACER 1.5 (Rambaut and Drummond 2009). We then tested whether Bayesian skyline plots were more appropriate than constant-size (Hudson 1990), exponential growth, or logistic growth models run with equivalent priors. The best model had the highest smoothed marginal likelihood (ln *L* ± SE, 1000 bootstrap pseudoreplicates) and was compared to alternatives using log Bayes factors (log_10_ *B*_10_) calculated in TRACER (Suchard et al. 2001), and established support criteria (Drummond et al. 2005). To complement our BEAST analyses, we estimated Ramos-Onsins and Rozas' (2002) *R*_2_ and Tajima's (1989) *D* neutrality statistics and their 95% confidence intervals using coalescent simulations in DnaSP (10^4^ replicates). To distinguish population expansions from purifying or positive natural selection, we tested for neutrality using similar simulations of Fay and Wu's (2000) *H* statistic. Agreement across methods (support for skylines showing expansions; positive, significant *R*_2_; negative, significant *D*; and nonsignificant *H* estimates) and taxa would provide strong evidence for congruent past population dynamics.

## Results

### Genetic diversity and neutrality

Among localities, mtDNA genetic diversity was highly spatially variable: *π* ranged from 0 to 0.0244, *Hd* ranged 0 to 1, and *θ*_w_ ranged 0 to 25.92 (Table S1). When averaged over local subpopulations, intraspecific *Hd* (range = 0.384–0.539; cross-species mean≈0.473), *π* (range = 0.0004–0.0076), and *θ*_w_ (range = 0.733–5.586) varied from low to moderate, but were much higher in *A. cultratus* and *P. gillii* than *X. umbratilis* (Table S1). MtDNA genetic diversity also varied greatly among population groups (see below): *π* ranged from 0.541 to 7.505, *Hd* ranged 0 to 0.944, and *θ*_w_ ranged 0.542 to 12.074 (Table 1). However, diversity peaked in groups located in the southeast of the study area in all three species. Haplotype diversity and *θ*_w_ peaked in the southeastern *P. gillii* population group, the southeastern *A. cultratus* group displayed the highest *π*, and *Xenophallus* diversity peaked in the lower San Juan-Tortuguero group (Table 1; see group designations below). Likewise, genetic diversity was higher within back-arc drainages than the San Juan, although local *A. cultratus* subpopulations had slightly higher mean intradrainage diversity relative to the other taxa (Table S3). Cyt*b* variation met expectations of neutral evolution in all three species (HKA test: *A. cultratus χ*^2^ = 0.004, *P* = 0.951; *P. gillii χ*^2^ = 0.256, *P* = 0.613; *Xenophallus χ*^2^ = 0.244, *P* = 0.622).

**Table 1:** Summary statistics and neutrality test results for *Alfaro cultratus*, *Poecilia gillii*, and *Xenophallus umbratilis* and homogeneous populations inferred within species using BARRIER

Parameter	*A. cultratus*			*P. gillii*				*Xenophallus*					
		
	NWG	SEG	All	NWG	FRPOG	SEG	All	NWG	UG	LSJTOG	COG	PAG	All
*N*	147	208	355	16	24	103	143	24	61	30	8	8	131
No. localities	8	12	20	2	2	15	19	2	13	6	1	2	24
*S*	44	52	58	6	11	93	98	4	9	20	0	2	96
*h*	20	33	46	5	8	29	37	4	9	12	1	3	29
*Hd* (±SE)	0.811 (±0.021)	0.880 (±0.016)	0.924 (±0.007)	0.717 (±0.095)	0.707 (±0.082)	0.939 (±0.010)	0.946 (±0.007)	0.424 (±0.113)	0.404 (±0.079)	0.892 (±0.031)	0.000 (±0.000)	0.679 (±0.122)	0.843 (±0.027)
*π*	0.0063	0.0125	0.0217	0.0018	0.0012	0.0101	0.0093	0.0005	0.0005	0.0061	NA	0.0007	0.0286
*θ*_w_	0.0132	0.0146	0.0150	0.0016	0.0026	0.00157	0.0155	0.0009	0.0017	0.0044	NA	0.0007	0.0155
*R*_2_	0.086*	0.081*	0.075*	0.155*	0.139*	0.089*	0.087*	0.156 ns	0.116*	0.119 ns	NA	0.241 ns	0.088*
Tajima's *D*	−0.082 ns	−0.112 ns	−0.119 ns	−0.058 ns	−0.046 ns	−0.098 ns	−0.117 ns	−0.027 ns	−0.017 ns	−0.080 ns	NA	−0.018 ns	−0.110 ns
Fay and Wu's *H*	−0.032 ns	−0.034 ns	−0.027 ns	0.028 ns	−0.009 ns	−0.064 ns	−0.063 ns	0.002 ns	−0.001 ns	−0.076 ns	NA	0.018 ns	0.323 ns

NA, not available; ns, not significant.

Significant results are shown in bold (*P < 0.01).

### Overall spatial incongruence

Overall, mtDNA analyses recovered incongruent spatial-genetic structuring among the three focal species in this study. The best SAMOVA grouping schemes partitioned the sampling area into two *A. cultratus* groups (*K* = 2, Φ_*CT*_ = 0.709), six *P. gillii* groups (*K* = 6, Φ_*CT*_ = 0.543), and seven *Xenophallus* groups (*K* = 7, Φ_*CT*_ = 0.973), indicating differing numbers and positions of spatial subdivisions between homogeneous populations within species (Figs. S1, S2, and Appendix S2). Genetic barriers detected using Monmonier's algorithm were similar but not identical to SAMOVA results; for example, BARRIER yielded two *A. cultratus* groups that were identical to those from SAMOVA, but fewer *P. gillii* and *Xenophallus* groups (Fig. 3 and Table 2). Still, grouping schemes resulting from both methods yielded matching barriers within each species, and were supported by independent AMOVAs (Table 2). Together, these results suggested that a significant barrier to gene flow (mean BP = 88.2%, across five segments) divided *A. cultratus* range into northwestern (NWG) and southeastern groups (SEG) in the lowlands between Rio Frio and Rio Pocosol flanked by Tenorio and Arenal volcanoes (Fig. 3A). This barrier separated Lake Nicaragua tributaries (e.g., Rio Frio) from others, including the nearby San Carlos basin where we sampled all three taxa at site 8 (Fig. 2 and Table S1). In *P. gillii*, a similar well-supported break (mean BP = 79.3%, six segments) occurred just east of Rio Pocosol, dividing Frio and Pocosol rivers subpopulations (FRPOG) from a southeastern group (SEG) of lower San Juan and Rio Tortuguero samples (Fig. 3B), including the San Carlos site. A strongly supported *P. gillii* break (BP = 97%) between Sabalo and Frio rivers formed a northwestern group (NWG) of westernmost Lake Nicaragua-tributary subpopulations (Rio Sapoa, Rio Sabalo). In *Xenophallus* (Fig. 3C), three barriers delimited an upland group (UG) confined to upper San Carlos tributaries separated from a large group localized in low-elevation San Juan and Rio Tortuguero tributaries (LSJTOG), and two smaller groups confined to the Corinto (COG; Rio Chirripó drainage) and Parismina rivers (PAG). The UG–LSJTOG barrier separated higher-elevation *Xenophallus* sites from low-elevation ones. Combined, SAMOVA and BARRIER results supported a similar pattern delimiting a fifth, northwestern *Xenophallus* group (NWG) divided from the other groups just northwest of the active Arenal volcano within the Guanacaste Cordillera highlands (Fig. 3C).

**Table 2:** AMOVA tests of models reflecting the best grouping schemes inferred using SAMOVA and BARRIER, plus two a priori biogeographical hypotheses of hierarchical genetic structuring within/among drainages

Comparison (number of groups)	Source of variation (percentage)			Φ-statistics		
	
	Among groups	Among subpopulations, within groups	Within subpopulations	Φ_*CT*_	Φ_*SC*_	Φ_*ST*_
*Alfaro cultratus*						
SAMOVA / BARRIER model (*K* = 2)	70.9	9.0	20.1	0.71**	0.31**	0.80**
1. Rio San Juan vs. back-arc drainages (2)	17.7	54.9	27.4	0.18 ns	0.67**	0.73**
2. Rio San Juan trib. by trib. (6)	15.8	58.7	25.5	0.16 ns	0.70**	0.75**
*Poecilia gillii*						
SAMOVA model (*K =* 6)	54.3	13.7	32.0	0.54**	0.30**	0.68**
BARRIER model (*K =* 3)	17.8	47.4	34.8	0.18*	0.58**	0.65**
1. Rio San Juan vs. back-arc drainages (2)	22.1	44.0	33.9	0.22**	0.56**	0.66**
2. Rio San Juan trib. by trib. (6)	−24.1	79.0	45.0	−0.24 ns	0.55**	0.64**
*Xenophallus*						
SAMOVA model (*K* = 7)	97.3	1.4	1.3	0.97**	0.52**	0.99**
BARRIER model (*K* = 5)	95.0	4.1	0.9	0.95**	0.82**	0.99**
1. Rio San Juan vs. back-arc drainages (2)	22.7	76.2	1.2	0.23 ns	0.99*	0.99*
2. Rio San Juan trib. by trib. (5)	93.0	6.0	1.0	0.93**	0.86**	0.99**

ns, not significant.

“Comparisons” are models (trib., tributary) and “number of groups” corresponds to population groups compared under each model. Sources of variation are percentages representing hierarchical partitioning of diversity across levels (negative percentages are interpreted as not significantly different from zero), and Φ-statistics range from 0, indicating no genetic structure, to 1, indicating complete isolation. Φ_*CT*_ is the correlation of random haplotypes within a group relative to the whole dataset (i.e., among groups), with significant results bolded (see Appendix S2 for further details).

*P < 0.05; **P < 0.01.

Mantel tests supported isolation-by-distance in *Xenophallus* (normalized Mantel coefficient = 0.307, *t =* 3.512, *P* = 0.003) but not the other species (*A. cultratus*: normalized Mantel coefficient = 0.0394, *t* = 0.464, *P* = 0.320; *P. gillii*: normalized Mantel coefficient = 0.138, *t =* 1.428, *P* = 0.0752). Likewise, *Xenophallus* genetic and ln-geographic distances showed a positive regression relationship not recovered in *A. cultratus* or *P. gillii* (Fig. S3).

Based on biogeographical AMOVA results, we rejected congruent genetic structuring across taxa within and among a priori drainage groups and San Juan tributary drainages (Table 2). Consistent with gene flow of alleles among demes in different drainages, *A. cultratus* showed nonsignificant genetic structuring among San Juan–back-arc drainage groups (AMOVA model 1) and *A. cultratus* and *P. gillii* had nonsignificant structuring among San Juan tributaries (AMOVA model 2). However, *P. gillii* were significantly differentiated between San Juan and back-arc drainage groups. In contrast, *Xenophallus* AMOVA 2 indicated San Juan tributaries were distinct from one another, but that San Juan–back-arc drainage structuring was nonsignificant (Table 2).

Gene tree analyses further highlighted phylogeographic incongruence. *Alfaro cultratus* was monophyletic with three well-supported clades (Figs. 3A, S4) diverged on average 3.2% at cyt*b*. *Alfaro cultratus* clades were mostly overlapping mosaics of subpopulations that mapped poorly to drainages but closely matched population groups inferred using SAMOVA/BARRIER: with few exceptions, haplotypes comprising clades I–III were confined to the SEG group, while those of clade IV fell into the NWG. Located respectively west versus east of the NWG–SEG barrier, clades I and IV were maximally diverged (4.2%). Clades III and IV had star-like networks with ancestral Sapoa, San Carlos, and Sixaola drainage haplotypes possibly indicating recent population expansions in these regions, and we estimated a San Carlos origin for the network root (haplotype 1). *Poecilia gillii* (Figs. 3B, S4) displayed two geographically overlapping mtDNA clades, limited spatially isolated or genetically distinct variation, ∼2–4% divergences at cyt*b* (max. divergence: 4.6%, clades I versus II), and small well-supported San Juan and back-arc drainage subclades (subclade II-a: haplotypes 38–39, mainly San Carlos; II-b: 40–42, mainly Sapoa, Sabalo and Frio; and II-c: 59–61, Matina). Haplotype 24 (San Carlos) was ancestral, sister to all other *P. gillii* haplotypes; however, haplotype 62 (Parismina) was the network root and showed a star-like pattern consistent with recent expansion. While the SEG harbored most *P. gillii* alleles, haplotypes 13 and 38–41 were confined to the NWG group. *Xenophallus* differed from the other species in having four well-supported, nonallopatric clades mostly isolated in drainage basins. The *Xenophallus* cyt*b* topology contrasted deep (6.0%) divergence of San Carlos (clade I) haplotypes (e.g., haplotype 29, the network root), from all others, against shallow intradrainage variation (Figs. 3C, S4). As in the other species, *Xenophallus* gene tree and network results also supported the genetic barriers inferred in BARRIER, with UG samples largely constituting clade I, COG and PAG samples largely constituting clade II, and clade III presenting a mixture of NWG, UG, and LSJTOG haplotypes. A star-like network pattern was only recovered among *Xenophallus* UG haplotypes, consistent with intradrainage expansion (Fig. 3C).

### Finer-scale spatial congruence

Whereas the above results indicated overall spatial incongruence, congruent genetic structuring was supported over finer spatial scales in one area. SAMOVA, BARRIER, and phylogenetic results revealed genetic differentiation in the same subregion of northwestern Costa Rica in all three species, with common differentiation just west of the San Carlos basin or between lowland-to-upland Frio and San Carlos sites, but all along the western edge of the San Carlos (Figs. 3, S1, S4). The pertinent breaks split the *A. cultratus* NWG–SEG, *Xenophallus* UG–LSJTOG, and *P. gillii* FRPOG–SEG groups (Fig. 3). These “northwest Costa Rica breaks” corresponded to species main pairwise population divergences, including BP-supported barriers, or barriers with the highest TrN distances identified in BARRIER.

### Temporal incongruence

Coalescent-based dating analyses in IMa2 yielded reliable estimates of BARRIER population group sizes (*θ*) and divergence times (*t*) in most runs, indicated by likelihood surface peaks (Fig. 3, Table 3, and Appendix S3). Obtaining confidence intervals for *t* was difficult, however, because some runs peaked at lower values before converging to positive values at larger *t*, representing infinite migration; thus, we accepted likelihood peaks as the best parameter estimates. So, although we found congruent northwestern Costa Rica spatial breaks, peak posterior *t* estimates revealed temporal incongruence for the three species overall and at the shared break (Fig. 3 and Table 3). Miocene–mid-Pleistocene divergences in northwest Costa Rica were much more likely in *A. cultratus* (NWG–SEG: *T*_div_ range = 3.583–1.612 Ma) and *Xenophallus* (UG–LSJTOG: *T*_div_ range = 13.731–2.334 Ma) than *P. gillii*. All divergences within *P. gillii* ranged over mid-late Pleistocene, including the FRPOG–SEG population pair (*T*_div_ range = 0.130–0.0221 Ma). A similar pattern of incongruence arose when comparing all *T*_div_ estimates together. Whereas we estimated nonzero migration rates in *A. cultratus*, posterior *m* distributions peaked at the lower limit of resolution or 95% HPDs included zero in *P. gillii* and *Xenophallus*, indicating no ongoing gene flow.

**Table 3:** Coalescent divergence time analysis parameter estimates

Species	Comparison (1–2)	*θ*_1_	*θ*_2_	*m*_1→2_	*m*_2→1_	*t*	*T*_div_, 2% rate (Ma)	*T*_div_, 0.9% rate (Ma)
*Alfaro cultratus*	NWG–SEG	14.920	39.560	0.348	0.166	9.690	1.612	3.583
	95% HPDs	7.720, 24.280	27.160, NA	0.108, 0.923	0.178, 0.438	3.013, NA	0.953, NA	2.119, NA
*Poecilia gillii*	NWG–SEG	6.750	53.25	0.0005	0.528	1.278	0.112	0.659
	95% HPDs	1.750, NA	29.250, 85.750	0.000, NA	0.257, NA	–	–	–
	NWG–FRPOG	7.350	5.850	0.00171	0.341	1.468	0.129	0.757
	95% HPDs	1.770, 25.410	1.410, 18.570	0.000, 2.359	0.000, 2.349	0.755, NA	0.0662, NA	0.390, NA
	FRPOG–SEG	4.750	45.250	0.000	0.000	0.253	0.0221	0.130
	95% HPDs	1.250, 14.250	22.250, 82.250	–	–	0.0975, 0.548	0.0009, 0.0480	0.050, 0.283
*Xenophallus*	UG–LSJTOG	9.750	29.750	0.000	0.000	26.610	2.334	13.731
	95% HPDs	3.750, 21.750	16.250, 49.750	–	–	5.850, 35.550	0.513, 3.118	3.019, 18.344
	LSJTOG-PAG	29.500	3.500	0.000	0.000	11.140	0.977	5.748
	95% HPDs	14.500, 50.500	0.000, NA	–	–	4.537, NA	0.398, NA	2.341, NA
	COG–UG	0.500	9.500	0.0004	0.0004	2.138	0.188	1.103
	95% HPDs	0.000, 11.500	3.500, 23.500	0.000, 0.6764	0.000, 0.301	0.613, NA	0.0537, NA	0.316, NA
	COG–LSJTOG	0.250	28.750	0.000	0.000	10.190	0.894	5.258
	95% HPDs	0.000, NA	16.250, 49.250	–	–	4.388, 16.860	0.385, 1.479	2.264, 8.700

Estimates of population sizes (*θ*_1_, *θ*_2_); migration rates (*m*); mutation-scaled population divergence times (*t*); and absolute divergence times (*T*_div_) in millions of years ago, inferred in IMa2 are shown for pairwise comparisons of diverged population groups (regions) from BARRIER (see Results, Fig. 3). Estimates were similar across three final runs using different random seeds, so results from best runs are presented. In brackets, 95% highest posterior density intervals (HPDs) are given where complete posterior distributions appeared to be estimated; whereas bounds that could not be estimated are listed as not available (NA), and zeros (with no density intervals) are given for *m* estimates in models for which pilot runs recovered zero migration hence *m* priors were set to zero in final runs. We calculated *T*_div_ using different mutation rates (*μ*), including the standard 2% rate for vertebrate mtDNA genes (Brown et al. 1979; Wilson et al. 1985), and a more slowly evolving 0.9% salmonid mtDNA rate (Martin and Palumbi 1993).

Akin to IMa2 results above, tests for simultaneous diversification at a finer-scale level within northwestern Costa Rica using approximate Bayesian computation models also revealed a striking pattern of temporal incongruence. MTML-msBayes results were nearly identical across four models with slightly different priors; therefore, we present results from one representative model (M2, upper *θ =* 0.01, upper *E*[*τ*] = 2, *Nm* = 0), though prior settings and results for all models can be found in Table S4. The *Ψ* (mean = 2.291) and *Ω* (mean = 0.269, 95% HPD range = 0.000–0.657; Bayesian posterior probability of one divergence event from polychotomous regression = 0.149) parameter estimates indicated that a model of multiple discrete divergences, rather than simultaneous divergence, was supported by the data (Fig. 3 and Table S4). This was also supported by hypotheses testing: based on Bayes factors of 4.303 for *Ψ* > 1 versus *Ψ* = 1, the data provide substantial support for a model with multiple divergences. In contrast, Bayes factors of 0.930 and 0.947 indicated only marginal weight of evidence (Jeffreys 1961) for simultaneous divergence (*Ψ* = 1 vs. *Ψ* > 1) and continuous divergence (*Ψ* = 3 vs. *Ψ* < 3) respectively. Based on a Bayes factor of 1.535 for *Ω* > 0.01 vs. *Ω* < 0.01, dispersion index *Ω* also indicated evidence against a simultaneous divergence model; however, the weight of the evidence was marginal, suggesting a potentially weaker ability of *Ω* to reject simultaneous divergence for our data. Modal divergence time estimates across the three population pairs from MTML-msBayes were similar to *T*_div_ estimates from IMa2 falling mostly within the Pliocene–Pleistocene (Table S4).

### Historical-demographic incongruence

The *P. gillii* data provided substantial support for broadly incongruent historical demography. The data supported the *P. gillii* Bayesian skyline plot over the other competing demographic models based on Bayes factors (Table 4), and plotting the skyline reconstruction of population dynamics through time revealed *P. gillii* late Pleistocene growth following slight population bottlenecking ∼40 ka (Fig. S5). *Poecilia gillii* population expansion was also supported by significant (*P* < 0.01) and positive *R*_2_ statistics (Table 1), as well as a star-like pattern of haplotypes radiating from the network root (Fig. 3B); however, the expansion signal was not recovered by Tajima's *D*, which was negative but nonsignificant. *Alfaro cultratus* results were intermediate to those of *P. gillii*: whereas Bayes factors strongly supported the constant model over the other competing models (Table 4), significant and positive *R*_2_ statistics supported expansions overall and within *A. cultratus* population groups despite negative and nonsignificant Tajima's *D* values (Table 1). This was surprising, given parsimony networks showed evidence for finer-scale *A. cultratus* expansions within regions (see above, Fig. 3A). Contrasting patterns in the other taxa, essentially all *Xenophallus* results pointed to a constant population size over time. Bayes factors less than 0.5 indicated that *Xenophallus* models were indistinguishable (Jeffreys 1961); thus, by parsimony, the constant model (model with the fewest parameters) is the most likely best-fit *Xenophallus* model. Consistent with population stasis, most *Xenophallus* networks suggested stable population structuring, and most groups had nonsignificant *R*_2_ and Tajima's *D* values (Fig. 3B and Table 1). However, the finer-scale *Xenophallus* UG population expansion in the San Carlos basin revealed by the network (above) was supported by a positive and significant *R*_2_ (Table 1). Results of Fay and Wu's *H* tests supported a scarcity of high frequency variants suggesting that the historical demographic inferences above do not reflect purifying or positive natural selection (*P* > 0.05; Table 1). Demographic models in Beast yielded intraspecific *t*_MRCA_s that were comparable to IMa2 and MTML-msBayes estimates, peaking ∼1.9–1.4 Ma around early Pleistocene, with overlapping Miocene–mid-Pleistocene confidence intervals (Table 4).

**Table 4:** Bayes factor tests comparing Bayesian coalescent demographic models

Species	Model	*t*_MRCA_ (Ma)	Smoothed ln likelihood (L) ± SE	Bayes factors (log_10_ *B*_10_)			

				BSP	Constant	Exponential	Logistic
*Alfaro cultratus*	BSP	1.358 [0.448, 4.193]	−1508.083 ± 0.188	–	−1.586	−1.793	−1.826
	**Constant**	1.398 [0.460, 4.272]	−1504.432 ± 0.185	1.586*	–	−0.207	−0.240
	Exponential	1.086 [0.423, 2.863]	−1503.955 ± 0.186	1.793*	0.207	–	−0.033
	Logistic	1.329 [0.449, 4.061]	−1503.879 ± 0.159	1.826*	0.240	0.033	–
*Poecilia gillii*	**BSP**	1.937 [0.677, 5.859]	−2335.298 ± 0.119	–	1.978*	1.682*	1.971*
	Constant	1.842 [0.656, 5.605]	−2339.853 ± 0.135	−1.978	–	−0.296	−0.006
	Exponential	1.472 [0.628, 3.827]	−2339.172 ± 0.137	−1.682	0.296	–	0.289
	Logistic	1.740 [0.633, 5.219]	−2339.838 ± 0.134	−1.971	0.006	−0.289	–
*Xenophallus*	BSP	1.937 [0.677, 5.859]	−2263.109 ± 0.094	–	0.008	−0.208	0.079
	**Constant**	1.842 [0.656, 5.605]	−2263.128 ± 0.099	−0.008	–	−0.216	0.071
	Exponential	1.597 [0.735, 4.217]	−2262.630 ± 0.093	0.208	0.216	–	0.287
	Logistic	1.823 [0.716, 5.507]	−2263.291 ± 0.092	−0.079	−0.071	−0.287	–

Geometric mean *t*_MRCA_ estimates based on sufficient MCMC-chain mixing in Beast (ESS > 400) are shown in millions of years ago with 95% HPDs in brackets, followed by ln-likelihood estimates from Tracer (±standard error [SE]). Bayes factors are presented as row-by-column comparisons. Best-fit models based on Jeffreys' (1961) “weight of evidence” criteria (*strong support) are presented in bold.

## Discussion

**Figure 4:**
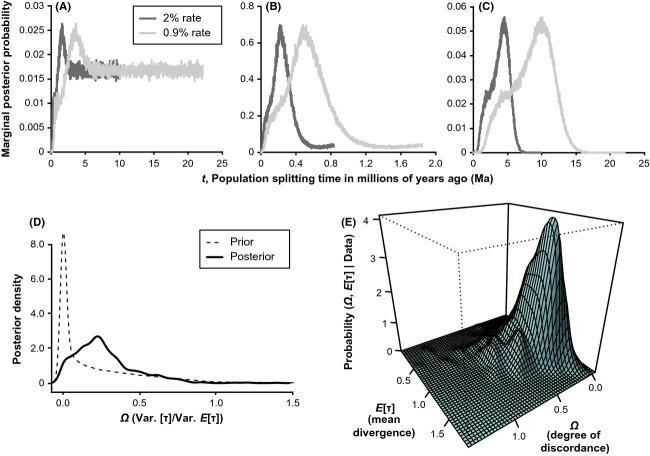
Posterior distributions and temporal incongruence of divergence times among northwest Costa Rican population pairs. The first three panels display marginal posterior probabilities of *t* parameters estimated by IMa2 for *Alfaro cultratus* (A), *Poecilia gillii* (B), and *Xenophallus umbratilis* (C), with probabilities estimated from two separate substitution rates. The two lower panels present results from MTML-msBayes analyses, including a comparison of the prior distribution versus the posterior densities of the number of divergence times across population pairs, *Ψ* (D), as well as a surface plot of the joint posterior probability densities of *E*[*τ*] (E).

The paradigm view in historical biogeography holds that congruent spatial-genetic subdivisions among codistributed taxa are most parsimoniously explained by a shared biogeographic history, whereas spatially incongruent patterns reflect independent responses owing to biological differences (Arbogast and Kenagy 2001; Avise 2000; Bermingham and Martin 1998; Hickerson et al. 2010; Sullivan et al. 2000; Donoghue and Moore 2003; Feldman and Spicer 2006; Bagley and Johnson 2014; refs. therein). Moreover, temporally incongruent patterns are thought to reflect multiple divergences in response to different events (Cunningham and Collins 1994; Donoghue and Moore 2003). Thus, a key question in historical biogeography is whether testing for shared biogeographic history supports concerted, independent, or multiple evolutionary responses. From empirical tests for spatial and temporal phylogeographic congruence among three livebearing fish species from the Nicaraguan depression of Central America, we find considerable evidence that the evolution of these taxa has not been concerted. Instead, these fishes display strikingly incongruent spatial-genetic structuring (Figs. 3, S1–S4, Appendix S2, and Table 2) *and* temporal population divergences (Fig. 4, Tables 3, 4, S4)–an overall pattern of pseudoincongruence. We therefore reject the concerted- and independent-response hypotheses. Our results suggest that our focal species have neither responded *solely* in lockstep fashion nor *solely* individualistically to long-term effects of shared biogeographic history, but that multiple geological or climatic events within the complex Nicaraguan depression landscape have shaped their population structuring. Multiple responses during recent community assembly involving different geographical distributions or colonization routes appear to have played a role in shaping the phylogeographic and community composition of the northern lower Central American freshwater fish assemblage. While drawing more robust conclusions about the precise number and underlying causes of population divergences inferred herein using mtDNA will require additional data from multiple unlinked nuclear loci, our study represents an important first step toward unraveling the history of the fish communities in this region. Indeed, ours is the first comparative analysis establishing a geographical and temporal framework for understanding diversification of northern LCA freshwater biota. Our results also provide some evidence that multiple evolutionary responses across these species were overlaid by incongruent demographic histories (Tables 1, 4 and Fig. S5). Here, we explore each level of incongruence among our results–temporal, spatial, and demographic, as well as ecological factors that, in addition to a multiple-response scenario, potentially explain the patterns we observed.

Statistical phylogeography studies have repeatedly underscored the importance of testing for temporal congruence while accounting for potentially confounding factors influencing divergence time estimates, such as mutational and coalescent gene-tree stochasticity (e.g., Edwards and Beerli 2000; Wakeley 2003; Nielsen and Beaumont 2009). We empirically tested for temporal congruence across multiple codistributed species while using methods that explicitly model isolation processes, demographic events, and coalescent variance, for example, Bayesian simulations sampling many coalescent gene genealogies. At the broadest temporal scales relevant for our data (i.e., thousands to millions of years), we inferred Miocene–mid-Pleistocene divergences between regions in *A. cultratus* and *X. umbratilis*, but mid-late Pleistocene divergences across barriers in *P. gillii* (Fig. 3 and Table 3), using full-Bayesian IMa2 analyses. Their different timescales of diversification are in greatest accord with the interpretation that these ND livebearing fishes represent possibly ancient but asynchronously evolved lineages that did not disperse into the study area at the same time. In other words, a parsimonious explanation of these patterns is that these species had different past distributions, thus experienced different dispersal and vicariance events at different times. Our results preclude a scenario of ancient dispersal or vicariance in *P. gillii*, however, because regional divergence estimates agree with a possibly recent origin of this species in the study area. This is consistent with substantial evidence for a late Pleistocene bottleneck-expansion event in *P. gillii* (Fig. S5 and Tables 1, 4) that may have occurred during recent recolonization, or post-colonization expansion. Nevertheless, the well-known limitations of single-locus phylogeography studies warrant careful consideration of the effects of potential sources of uncertainty while interpreting these results. In particular, it is difficult to estimate demographic parameters from mtDNA, from which we should expect wider confidence intervals reflecting (1) the inherent stochasticity of coalescent processes (Hudson 1990; Edwards and Beerli 2000) and (2) the influence of varying levels of *N*_e_ or *m* across ancestral populations (Wakeley 2003; Nielsen and Beaumont 2009). While our IMa2 runs converged for most parameter estimates and estimated error in the inferences, chance events have likely influenced our results. Estimating confidence in population divergence times was problematic and gave broad, overlapping confidence intervals (Table 3). Moreover, approximately flat likelihood surfaces yielded large ancestral population size estimates, possibly indicating retained polymorphisms or that gene flow occurred between the ancestral population and other populations not included in the simple 2-population IMa2 models we employed. However, independent estimates of divergence times converged on similar results supporting conclusions drawn from the IMa2 analyses: multi-population coalescent models (i.e., Bayesian skyline plots) inferred intraspecific *t*_MRCA_s whose confidence intervals bracketed the majority of the IMa2 *t*-estimate probability densities, and we also estimated similar Pliocene–Pleistocene regional divergences in MTML-msBayes (Fig. 3 and Tables 4, S4). Hypotheses tests using Bayes factors to compare divergence models based on approximate Bayesian computation simulations also provided moderate to strong support for temporal incongruence (Table S4). Thus, despite potential issues with mtDNA time estimates, different methods support the inferred pattern of multiple evolutionary responses over a Miocene–Pleistocene timescale of diversification.

Qualitatively similar patterns of idiosyncratic temporal divergences have been reported in other comparative phylogeographic studies, including analyses of three codistributed freshwater fish lineages from southern LCA (Bermingham and Martin 1998; Reeves and Bermingham 2006), Mesoamerican rodents across the Isthmus of Tehuantepec (Sullivan et al. 2000), and California herpetofauna (Feldman and Spicer 2006). Our results therefore add to a growing body of evidence from different study systems worldwide supporting a commonality of temporally incongruent phylogeographic patterns in codistributed taxa. The divergence time estimates we report also closely approximate levels, thus the potential timing, of population divergences found in previous studies of lower Central American taxa. For example, a mtDNA-RFLP study of *Orthogeomys cherriei* pocket gophers found haplotypes were up to 1.5% diverged in the Costa Rican Central Cordillera (Demastes et al. 1996), which roughly correlates to mid-Pleistocene assuming the standard 2% pairwise vertebrate mtDNA rate (Bagley and Johnson 2014). A study of *Rhamdia guatemalensis* catfishes found that western Costa Rican populations isolated in the Rio Bebedero basin diverged from all other haplotypes just prior to the final closure of the LCA isthmus ∼3 Ma (Perdices et al. 2002). And multiple studies along the Panamanian Isthmus in southern LCA show that various lineages of electric knifefishes (Hypopomidae), seven-spine catfishes (Heptapteridae), and tetras (Characidae) also display Pliocene-late Pleistocene divergences similar to our findings (Bermingham and Martin 1998; Martin and Bermingham 2000; Reeves and Bermingham 2006). Combined with our results, these examples show that the relatively recent (∼7-0 Ma) geological history of emergent LCA isthmus lands (e.g., Fig. 1C) appears to have significantly constrained regional patterns and processes of evolutionary divergence, and this is consistent with a recent meta-analysis of divergence times reported in LCA phylogeography studies (Bagley and Johnson 2014).

Given we used the same locus (similar mutation rates) to compare the phylogeographies of closely related species with overlapping ranges, inadequate phylogenetic signal cannot account for the broad-scale pattern of spatial incongruence among *A. cultratus*, *P. gillii*, and *Xenophallus* (e.g., Figs. 3, S1). Instead, while our tests of temporal congruence show that the incongruent spatial-genetic subdivisions in these taxa arose during responses to different events at different times, other factors also potentially explain the observed spatial differences, including species-specific responses driven by different biological attributes (*cf*. Burney and Brumfield 2009; Fouquet et al. 2012). The livebearing fishes we studied share complex ecological adaptations for viviparity, benthopelagic habits, and nonsuperfetating reproduction (Winemiller 1993; Reznick and Miles 1989; Johnson and Bagley 2011; J. C. Bagley and J. B. Johnson, unpubl. data). Still, these species differ along key ecological axes indicating potentially superior dispersal propensity and wider physiological tolerances in *P. gillii* and *A. cultratus*, relative to *Xenophallus*. Most notably, *P. gillii* achieve larger maximum body size (105 mm; compared with 45–65 mm), a broader range of elevations (0–1220 m; compared with 0–590 m) and thermal environments (J. B. Johnson, pers. obs.), and a much larger geographic range (Guatemala through Panama, except southwestern Panama) than the other species (Bussing 1998; Smith and Bermingham 2005; but see Alda et al. 2013). *Alfaro cultratus* also display a much larger range (northern Nicaragua to western Panama) than *Xenophallus*, which is endemic to the study area (Bussing 1998) and is usually more abundant at the upper elevations of its range (J. C. Bagley and J. B. Johnson, pers. obs.). Furthermore, consistent with salinity tolerance and propensity for movement into peripheral habitats in other *Poecilia* (e.g., *P. mexicana*; Schlupp et al. 2002), *P. gillii* occur in brackish water, while our other focal species do not (Bussing 1998). These differences at ecological traits correlated to dispersal propensity, population size, and competitive ability have likely influenced unique phylogeographic signals in these species. Indeed, our results, combined with available ecological data, are consistent with the general prediction that phylogeographic structure within species should correlate inversely with behavioral preference or potential for dispersal (e.g., dispersal rates, distances) (Avise 2000; Bagley and Johnson 2014). This is best illustrated in *Xenophallus*, which displays evidence for relatively lower dispersal propensity, yet a higher degree of phylogeographic structuring indicated by deep phylogenetic divergences, haplotypes/clades mostly isolated in drainages (Figs. 3C, S4), and zero ongoing gene flow (Table 3) consistent with limited inter-drainage and -population migration. Mantel tests and regression analyses also supported isolation-by-distance only in *Xenophallus* (Fig. S3). Thus, landscape barriers and geographical distances have influenced phylogeographical structuring to a greater degree in *Xenophallus* than the other taxa most likely due to lower dispersal propensity. That *Xenophallus* AMOVA model 1 supported no structuring between the San Juan and Tortuguero rivers conflicts with this view (Table 2). However, this may reflect a recent stream capture event unrelated to dispersal ecology, or an artifact of limited *Xenophallus* sampling (*N* = 9) and genetic diversity (e.g., *S* = 2) within Rio Tortuguero (Table S1).

Population-level processes may also have contributed to the spatially incongruent subdivisions among the ND livebearers in this study. Because gene flow among demes, incomplete lineage sorting, and demographic fluctuations can produce similar genetic imprints (e.g., converging due to chance, or regional extinctions), teasing these processes apart is difficult. However, nonequilibrium statistical phylogeography tools such as IMa2 that jointly estimate demographic parameters while modeling coalescent and mutational stochasticity, implicitly test whether alleles shared between populations reflect gene flow versus incomplete lineage sorting (assuming uniform priors with *m* ≠ 0, as in all of our pilot IMa2 runs and several of our long runs). If marginal distributions of *m* parameters include 0, gene flow can be rejected in favor of incomplete lineage sorting; otherwise, there is sufficient information to resolve migration as influencing the data. In IMa2 we inferred discordant migration rates between regions among taxa, with nonzero migration between *A. cultratus* groups while other results suggested no ongoing gene flow (Table 3; Appendix S3). Thus shared alleles between diverged populations (Figs. 3, S4) are best explained by gene flow in *A. cultratus*, but appear more consistent with incomplete lineage sorting in the other taxa. While multiple unlinked loci are needed to obtain more accurate parameter estimates to test this initial mtDNA characterization, our migration estimates generally agree with the ecological context above (e.g., zero gene flow within presumably poorer dispersing *Xenophallus*). Moreover, we expect that samples from demes with higher *m* should be more polymorphic than those from demes with lower *m* (Wakeley and Aliacar 2001), and this is met by the polarized genetic variation displayed in *A. cultratus* (higher *π* and *Hd*) and *Xenophallus* (much lower *π* and *Hd*; Tables 1, S1). Zero-gene-flow inferences in *P. gillii* are exceptional to this (Table 3), as other data suggest this taxon may be a stronger disperser; however, low *m* estimates may indicate insufficient data for estimating migration in this species while fitting a six-parameter model. Alternatively, the small (<1) nonzero peak *m* estimates between *P. gillii* population groups (e.g., SEG and FRPOG into NWG) may simply indicate a trivial number of migrants relative to overall population size (Tables 1, 3).

Natural selection is another process with consequences for population genetic variation that can cause spatially incongruent phylogeographic breaks across codistributed species (Irwin 2002). However, while selection can play a role in shaping mtDNA genetic patterns (e.g., Machado and Hey 2003), it has unlikely influenced major patterns among our results. Coalescent simulations of neutrality test statistics demonstrate that our data conform to expectations of selectively neutral evolution (Table 1). Furthermore, we evaluated spatial phylogeographic congruence based on tests of whether genetic barriers were supported by bootstrapping (a randomization procedure) in BARRIER, which allows us to rule out a random pattern of barriers due to natural selection. Despite the utility of this approach, other studies drawing similar conclusions (e.g., Fouquet et al. 2012), have not evaluated this possibility; however, doing so seems more important in cases such as ours where broad-scale spatially incongruent patterns are recovered than in other cases.

In addition to temporally and spatially incongruent phylogeographic histories, we find evidence for incongruent patterns of historical-demographic fluctuations over recent timescales among ND livebearers (Figs. 3, S5 and Tables 1, 4). Our results support recent broad-scale expansion in *P. gillii* and overall stasis despite finer-scale expansions within regions in *A. cultratus* and *Xenophallus*. However, while Bayes factors strongly rejected the null model in *P. gillii* and strongly supported it in *A. cultratus*, they could not distinguish between skyline, constant, exponential and logistic demographic models in *Xenophallus*. Clearly, failing to reject the null model (size-constancy) provides a weaker basis for making inferences about past population dynamics than rejecting the null model would. However, size-constancy rather than bottleneck-expansions or other growth trends still appears to be the most likely historical demographic scenario for *Xenophallus* based on a parsimonious interpretation of Bayes factors (Table 4). The gene tree and network patterns (Figs. 3, S4) and neutrality test results (Table 1) also support size-constancy in *Xenophallus*.

Notwithstanding the many points of incongruence among our focal taxa, we have established that these species share genetic breaks just north of present-day Lake Arenal in northwestern Costa Rica, in between two large Rio San Juan tributaries, the Frio and San Carlos rivers (Figs. 1, 3). Considering the ecological and geological heterogeneity of LCA landscapes in and around the Nicaraguan depression (e.g., Fig. 1; reviewed in Bagley and Johnson 2014; Funk et al. 2009; Mann et al. 2007; Coates and Obando 1996; Coates et al. 2004), as well as the incongruent temporal, spatial, ecological, and demographic patterns discussed above, the fact that we find evidence for such congruent spatial breaks at such a fine spatial scale (<∼25 km) is rather astonishing. This spatial-genetic subdivision has also never been observed in other Costa Rican taxa aside from the livebearing fishes in this study (Bagley and Johnson 2014). Whereas paleoclimatic effects are often cited as the cause of phylogeographic breaks in terrestrial taxa (e.g., Avise 2000; Hewitt 2000), the geographical distributions of LCA freshwater fishes are principally controlled by drainage basin geomorphology and connectivity, (e.g., Bermingham and Martin 1998; Bussing 1998; Smith and Bermingham 2005). Thus, the observed Frio-San Carlos break most likely reflects a direct influence of different geological and sea level events on the drainage networks of the southern San Juan superbasin. As shown in Fig. 1C, LCA has experienced radical geological transitions and landscape changes (e.g., Coates and Obando 1996; Coates et al. 2004) and is physiographically defined by northwest-southeast-trending volcanic cordilleras of Quaternary age (e.g., Marshall et al. 2003; Marshall 2007). In the vicinity of the shared break, the upper reaches the Frio and San Carlos rivers and nearby drainages interact with the Guanacaste Cordillera; the Plio-Pleistocene activity of volcanoes within this part of the Central American volcanic arc seems most likely to have triggered vicariance or extinction-recolonization events responsible for the Frio-San Carlos break. Modern topography may also have contributed to a common pattern of genetic divergence between tributaries and drainages in this region, including the maintenance of isolation, for example as steep drainage gradients limited connectivity. Given evidence for finer-scale population expansions within the San Carlos basin in all three taxa, the present position of this barrier may reflect an ongoing process of secondary expansion following genetic drift in moderate to long-term isolation.

In summary, through multiple empirical tests for congruence, our study has demonstrated that spatially and temporally incongruent phylogeographic and demographic patterns are evident in three species of livebearing fishes that are codependent upon freshwater habitats within the Nicaraguan depression landscape. The majority of our results point to multiple evolutionary responses among these taxa, and we have statistically shown that these corresponded to multiple historical dispersal and vicariance events, possibly suggesting waves of dispersion through the area. Despite overall pseudoincongruence supporting a “multiple-response hypothesis”, however, landscape history appears to have promoted commonalities of phylogeographical structuring, albeit over fine spatial scales. More nuclear loci and expanded spatial sampling covering the entire species ranges are necessary to better tease apart the exact histories responsible for the varying evolutionary trajectories in these taxa. However, a comparative perspective has afforded us a view of the lower Central American freshwater fish assemblage that has provided insights into historical as well as ecological influences on population structure, and which permits drawing several future predictions. First, additional studies of individual taxa similarly confined to these freshwater habitats of the Nicaraguan depression should show similar phylogeographic patterns, although it is likely that even further evidence for a multiple-response scenario will be uncovered. And, secondly, we predict that additional comparative studies will yield many new insights into the relative roles of concerted, independent, and multiple responses in shaping the assembly and diversification of species rich and endemic Central American ecosystems.

## Supporting Information

**Figure S1.** SAMOVA maps with maximally genetically differentiated groups of samples separated by thick red lines. Dotted (thin) lines are Voroni diagram edges used during calculations. All models produced statistically significant fixation indices confirmed by independent AMOVAs (*P* < 0.001; Table 2).

**Figure S2.** Fixation index scores (Φ_*CT*_) from SAMOVA analyses plotted against the *K* value imposed during each run.

**Figure S3.** Regressions showing relationships between genetic distances and geographical distances [(ln) km] across all sampling sites (Fig. 2) for three species of LCA livebearing freshwater fishes.

**Figure S4.** “Best” maximum likelihood gene tree topologies with nodal support.

**Figure S5.** Bayesian skyline plots of effective population size changes. Historical skyline reconstructions of population size (*N*_e_*τ*, converted to *N*_e_ using generation time) through time for each species, correlated with the late Pleistocene eustatic sea level curve of (Lambeck et al. 2002; cited in the main text).

**Table S1.** Locality details, population group assignments, GenBank accession numbers, and DNA polymorphism levels across subpopulations.

**Table S2.** DNA substitution models selected using DT-ModSel.

**Table S3.** Cyt*b* DNA polymorphism levels within and among drainage basins.

**Table S4.** Model priors, estimated number and timing of divergence events, and Bayes factors from MTML-msBayes. Results are presented for four coalescent models (M1–M4) run in MTML-msBayes. Bayes factors were used to conduct hypotheses tests of posterior support for simultaneous divergence (e.g., *Ψ = 1*) versus other hypotheses.

**Appendix S1.** Sampling and outgroups details.

**Appendix S2.** SAMOVA and BARRIER methods and results.

**Appendix S3.** Coalescent divergence time estimation: IMa2 methods.
