# Supplementary figures and images for: Testing for shared biogeographic history in the lower Central American freshwater fish assemblage using comparative phylogeography: concerted, independent, or multiple evolutionary responses?
Source: Ecol Evol. 2014 Apr 10;4(9):1686–705. doi: 10.1002/ece3.1058 (PMC4063468; doi:10.1002/ece3.1058)

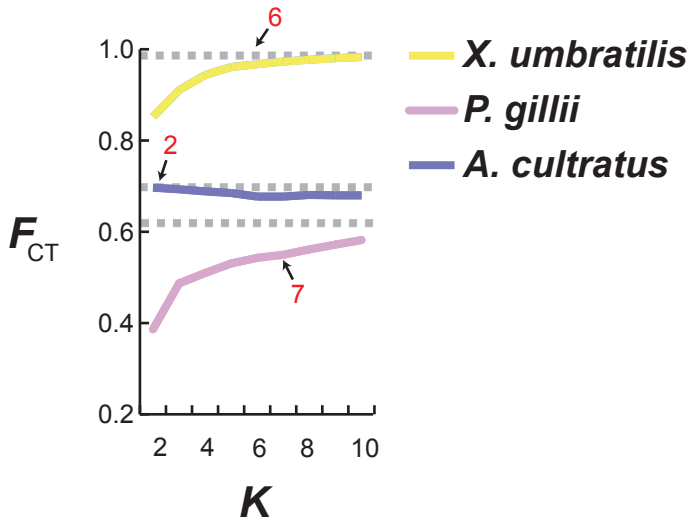

Supplement: Supplementary file 2 [file ece30004-1686-SD2.pdf]

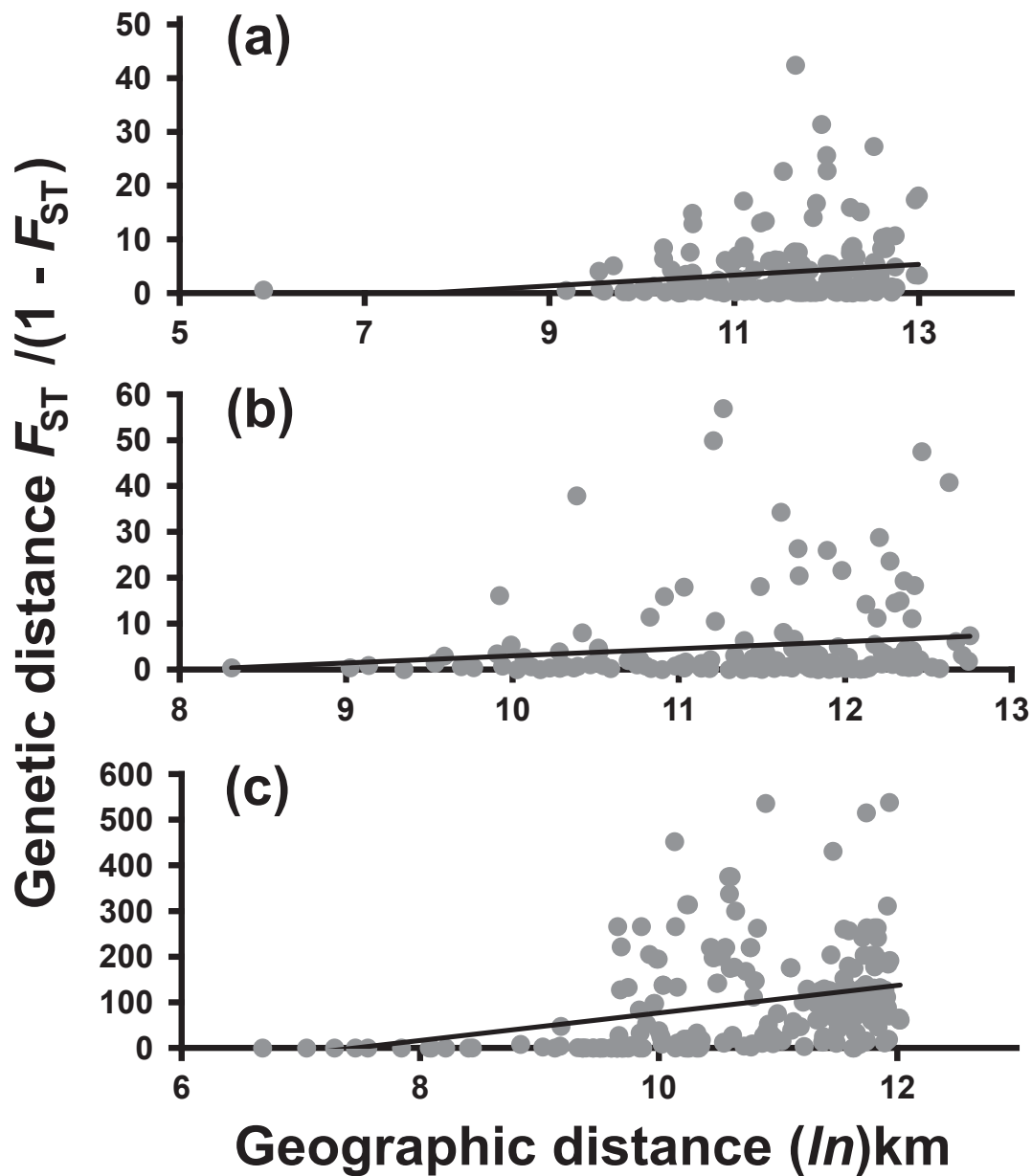

Supplement: Supplementary file 3 [file ece30004-1686-SD3.pdf]

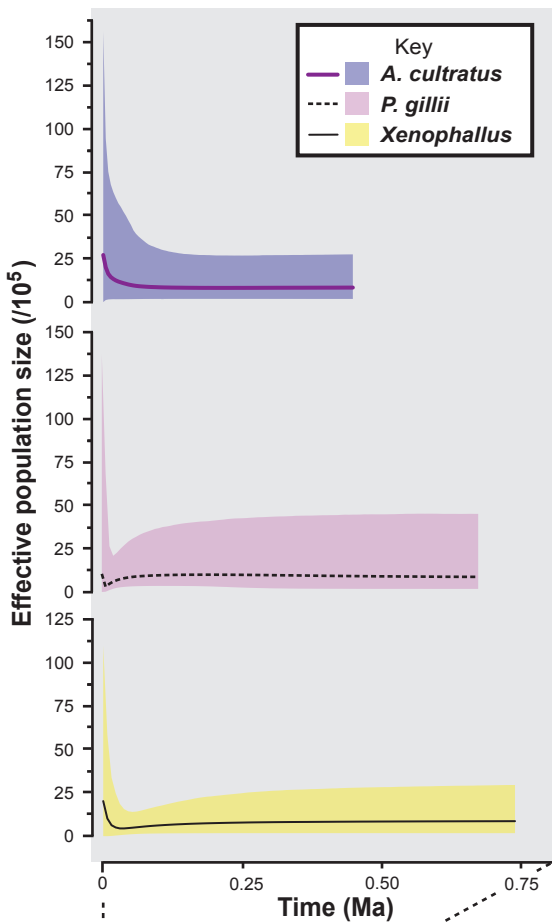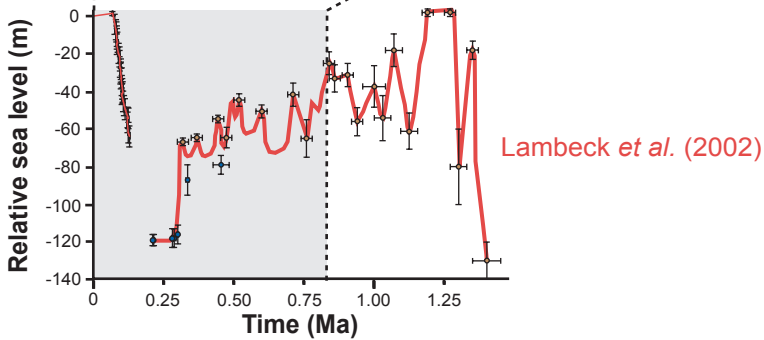

Supplement: Supplementary file 5 [file ece30004-1686-SD5.pdf]
