# Supplementary material for: Testing for shared biogeographic history in the lower Central American freshwater fish assemblage using comparative phylogeography: concerted, independent, or multiple evolutionary responses?
Source: Ecol Evol. 2014 Apr 10;4(9):1686–705. doi: 10.1002/ece3.1058 (PMC4063468; doi:10.1002/ece3.1058)

Fig. S1 'Best' ML topologies (observed gene trees) with nodal support.

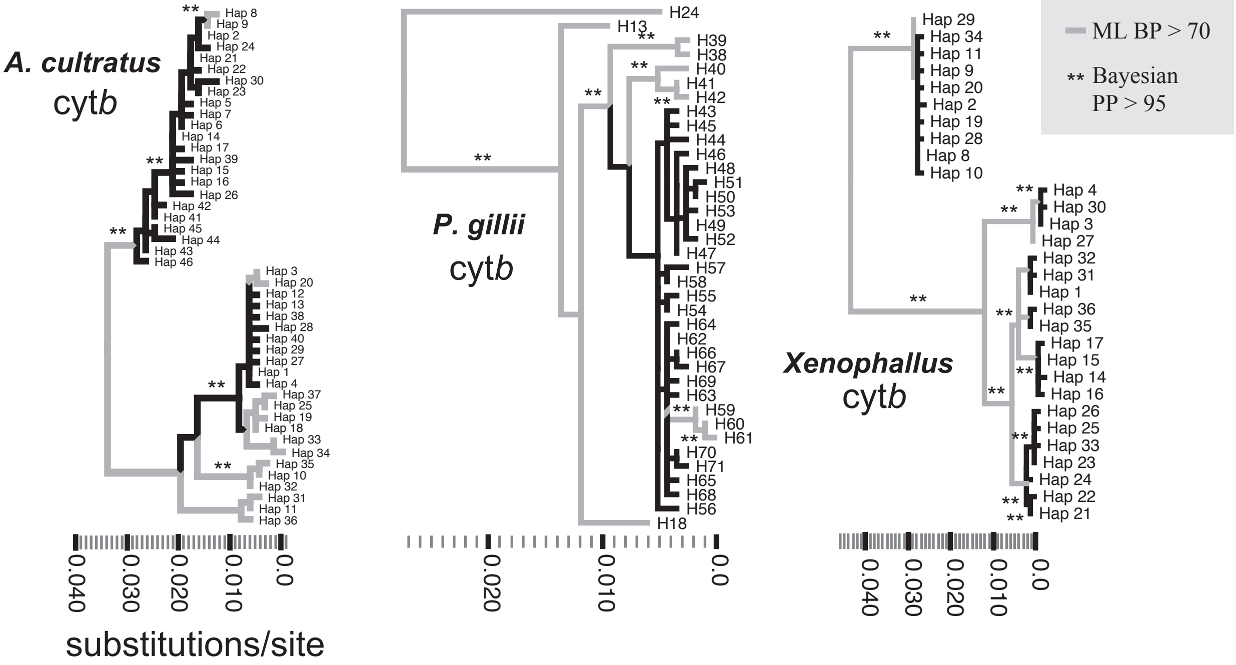

Supplement: Supplementary file 4 [file ece30004-1686-SD4.pdf]
