# Supplementary material for: Testing for shared biogeographic history in the lower Central American freshwater fish assemblage using comparative phylogeography: concerted, independent, or multiple evolutionary responses?
Source: Ecol Evol. 2014 Apr 10;4(9):1686–705. doi: 10.1002/ece3.1058 (PMC4063468; doi:10.1002/ece3.1058)
Supplement: Supplementary file 6 [file ece30004-1686-SD6.docx]

**Table S1 Locality details, population group assignments, GenBank accession numbers, and DNA polymorphism levels across sub-populations**

| **NOTE TO EDITOR: GenBank accession numbers for new sequence data generated in this study are pending (denoted "XXXXXXXX").** | | | | | | | | | | |  |  |  |  |  |  |  |
| --- | --- | --- | --- | --- | --- | --- | --- | --- | --- | --- | --- | --- | --- | --- | --- | --- | --- |
|  |  |  |  |  | **BARRIER** |  | **Lat.** | **Long.** |  | **GenBank nos.** | **Summary statistics** | | | | | | |
| **Species [Ref.]** | **Locality** | **Drainage** | **ID** | **CODE** | **population group** | ***N*** | **(°N)** | **(°W)** | **Country** | **cyt*b*** | *S* | *h* | *Hd* | s.e. *Hd* | π | *θ_w_* | *N* |
| ***Alfaro cultratus*** |  |  |  |  |  | **355** |  |  |  |  |  |  |  |  |  |  |  |
| *A. cultratus* [1] | NA | NA | — | — |  | 1 | — | — | Costa Rica | EF017531 | NA | NA | NA | NA | NA | NA | 1 |
| *A. cultratus* [2] | Lake Nicaragua (LN) | San Juan (1) | 1 | LN | NWG | 2 | 11.9240 | -85.9423 | Nicaragua | FJ178773, FJ178772 | NA | NA | NA | NA | NA | NA | 2 |
| *A. cultratus* [2] | Rio El Monje (Lake Managua, LM) | San Juan (1) | 2 | MONJE | NWG | 1 | 11.6333 | -86.3000 | Nicaragua | FJ178774 | NA | NA | NA | NA | NA | NA | 1 |
| *A. cultratus* (this study) | Rio Sapoa (Sapoa) | San Juan (1) | 3 | SAPF | NWG | 49 | 11.0444 | -85.6159 | Costa Rica | XXXXXXXX | 2 | 3 | 0.232 | 0.076 | 0.0004 | 0.453 | 47§ |
| *A. cultratus* (this study) | Rio Sabalo (Sabalo) | San Juan (1) | 4 | SABF | NWG | 54 | 11.0428 | -85.4892 | Costa Rica | XXXXXXXX | 31 | 7 | 0.335 | 0.082 | 0.0056 | 6.803 | 54 |
| *A. cultratus* (this study) | Rio Zapote (Zapote) | San Juan (1) | 5 | ZAPF | NWG | 12 | 10.8665 | -85.0339 | Costa Rica | XXXXXXXX | 2 | 3 | 0.439 | 0.158 | 0.0008 | 0.662 | 12 |
| *A. cultratus* (this study) | Rio Salto (Zapote) | San Juan (1) | 6 | SALF | NWG | 20 | 10.7982 | -85.0233 | Costa Rica | XXXXXXXX | 25 | 3 | 0.511 | 0.091 | 0.0047 | 7.047 | 20 |
| *A. cultratus* (this study) | Rio Venado (Frio) | San Juan (1) | 7 | VENF | NWG | 9 | 10.6448 | -84.8222 | Costa Rica | XXXXXXXX | 0 | 1 | 0.000 | 0.000 | 0.0000 | 0.000 | 9 |
| *A. cultratus* (this study) | Quebrada Perez (San Carlos) | San Juan (1) | 8 | PERF | SEG | 21 | 10.4735 | -84.8223 | Costa Rica | XXXXXXXX | 5 | 3 | 0.567 | 0.056 | 0.0022 | 1.390 | 21 |
| *A. cultratus* (this study) | Rio Chimurria (Pocosol) | San Juan (1) | 9 | CHIF | SEG | 27 | 10.7274 | -84.5582 | Costa Rica | XXXXXXXX | 29 | 6 | 0.708 | 0.066 | 0.0206 | 7.857 | 23§ |
| *A. cultratus* (this study) | Quebrada Piecueca (San Carlos) | San Juan (1) | 10 | PIEF | SEG | 7 | 10.3861 | -84.5790 | Costa Rica | XXXXXXXX | 27 | 3 | 0.524 | 0.209 | 0.0132 | 11.020 | 7 |
| *A. cultratus* (this study) | Rio Infernito (San Carlos) | San Juan (1) | 11 | INFF | SEG | 19 | 10.6180 | -84.4842 | Costa Rica | XXXXXXXX | 0 | 1 | 0.000 | 0.000 | 0.0000 | 0.000 | 19 |
| *A. cultratus* (this study) | Rio Caño Negro (San Carlos) | San Juan (1) | 12 | CANF | SEG | 4 | 10.3728 | -84.2782 | Costa Rica | XXXXXXXX | 2 | 2 | 0.500 | 0.265 | 0.0017 | 1.091 | 4 |
| *A. cultratus* (this study) | Rio Sarapiquí (Chirripó) | San Juan (1) | 13 | SARF | SEG | 20 | 10.5245 | -84.0313 | Costa Rica | XXXXXXXX | 24 | 7 | 0.753 | 0.079 | 0.0077 | 6.765 | 20 |
| *A. cultratus* (this study) | Rio Isla Grande (Chirripó) | San Juan (1) | 14 | ISLF | SEG | 19 | 10.3930 | -83.9682 | Costa Rica | XXXXXXXX | 7 | 5 | 0.591 | 0.118 | 0.0037 | 2.003 | 19 |
| *A. cultratus* (this study) | Rio Corinto (Chirripó) | San Juan (1) | 15 | CORF | SEG | 21 | 10.2119 | -83.8865 | Costa Rica | XXXXXXXX | 30 | 4 | 0.681 | 0.059 | 0.0096 | 8.339 | 21 |
| *A. cultratus* (this study) | Upper Río Tortuguero | Tortuguero (2) | 16 | TORS | SEG | 12 | 10.2594 | -83.8122 | Costa Rica | XXXXXXXX | 27 | 6 | 0.818 | 0.084 | 0.0162 | 8.941 | 12 |
| *A. cultratus* (this study) | Unnamed river | Parismina (2) | 17 | PARS | SEG | 20 | 10.1977 | -83.6521 | Costa Rica | XXXXXXXX | 7 | 4 | 0.575 | 0.115 | 0.0034 | 2.110 | 16§ |
| *A. cultratus* (this study) | Rio Herediana | Parismina (2) | 18 | PARS | SEG | 16 | 10.1242 | -83.5562 | Costa Rica | XXXXXXXX | 8 | 4 | 0.617 | 0.096 | 0.0049 | 2.411 | 16 |
| *A. cultratus* (this study) | Unnamed river | Sixaola (2) | 19 | SIXS | SEG | 19 | 9.6209 | -82.8577 | Costa Rica | XXXXXXXX | 33 | 7 | 0.854 | 0.043 | 0.0178 | 9.442 | 19 |
| *A. cultratus* (this study) | Rio Carbon | Sixaola (2) | 20 | SIXS | SEG | 3 | 9.6231 | -82.8552 | Costa Rica | XXXXXXXX | 22 | 3 | 1.000 | 0.272 | 0.0244 | 14.667 | 3 |
| **Means:** |  |  |  |  |  | **16.952** |  |  |  |  | **15.611** | **4** | **0.539** | **0.104** | **0.0076** | **5.056** | **16.476** |
|  |  |  |  |  |  |  |  |  |  |  |  |  |  |  |  |  |  |
| ***Alfaro huberi*** |  |  |  |  |  | **7** |  |  |  |  | — | — | — | — | — | — | — |
| *A. huberi* (this study) | — | Lis Lis | — | — | — | 2 | 15.6659 | -86.5802 | Honduras | XXXXXXXX | — | — | — | — | — | — | — |
| *A. huberi* (this study) | — | Cangrejal | — | — | — | 1 | 15.6530 | -86.0600 | Honduras | XXXXXXXX | — | — | — | — | — | — | — |
| *A. huberi* (this study) | — | Patuca | — | — | — | 2 | 14.8475 | -88.8749 | Honduras | XXXXXXXX | — | — | — | — | — | — | — |
| *A. huberi* (this study) | — | Motagua | — | — | — | 1 | 14.9054 | -89.1618 | Honduras | XXXXXXXX | — | — | — | — | — | — | — |
| *A. huberi* (this study) | Unnamed tributary | Unnamed tributary | — | — | — | 1 | 14.9978 | -89.1321 | Honduras | XXXXXXXX | — | — | — | — | — | — | — |
|  |  |  |  |  |  |  |  |  |  |  |  |  |  |  |  |  |  |
|  |  |  |  |  |  |  |  |  |  |  |  |  |  |  |  |  |  |
|  |  |  |  |  |  |  |  |  |  |  |  |  |  |  |  |  |  |
| ***Poecilia gillii*** |  |  |  |  |  | **143** |  |  |  |  |  |  |  |  |  |  |  |
| *P. gillii* [3] | Rio Sapoa (Sapoa) | San Juan (1) | 3 | PG725 | NWG | 8 | 11.0444 | -85.6159 | Costa Rica | — | 5 | 4 | 0.768 | 0.113 | 0.0020 | 1.928 | 8 |
| *P. gillii* [3] | Rio Sabalo (Sabalo) | San Juan (1) | 4 | PG726 | NWG | 8 | 11.0428 | -85.4892 | Costa Rica | — | 0 | 1 | 0.000 | 0.000 | 0.0000 | 0.000 | 8 |
| *P. gillii* [3] | Rio Venado (Frio) | San Juan (1) | 7 | PG719 | FRPOG | 16 | 10.6448 | -84.8222 | Costa Rica | — | 7 | 5 | 0.450 | 0.151 | 0.0008 | 2.110 | 16 |
| *P. gillii* [3] | Rio Sabalito (San Carlos) | San Juan (1) | — | PG608 | SEG | 5 | 10.5486 | -84.9808 | Costa Rica | — | 1 | 2 | 0.600 | 0.175 | 0.0005 | 0.480 | 5 |
| *P. gillii* [3] | Rio Chiquito (San Carlos) | San Juan (1) | — | PG612 | SEG | 5 | 10.4377 | -84.8682 | Costa Rica | — | 22 | 4 | 0.900 | 0.161 | 0.0109 | 10.560 | 5 |
| *P. gillii* [3] | Lake Arenal (San Carlos) | San Juan (1) | — | PG603 | SEG | 2 | 10.4721 | -84.7693 | Costa Rica | — | 12 | 2 | 1.000 | 0.500 | 0.0105 | 12.000 | 2 |
| *P. gillii* [3] | Rio La Palma (San Carlos) | San Juan (1) | — | PG602 | SEG | 5 | 10.4988 | -84.6890 | Costa Rica | — | 0 | 1 | 0.000 | 0.000 | 0.0000 | 0.000 | 5 |
| *P. gillii* [3] | Rio Infernito (San Carlos) | San Juan (1) | 11 | PG715 | SEG | 8 | 10.618 | -84.4842 | Costa Rica | — | 3 | 2 | 0.250 | 0.180 | 0.0007 | 1.157 | 8 |
| *P. gillii* [3] | Rio Chimurria (Pocosol) | San Juan (1) | 9 | PG716 | FRPOG | 8 | 10.7274 | -84.5582 | Costa Rica | — | 6 | 3 | 0.464 | 0.200 | 0.0013 | 2.314 | 8 |
| *P. gillii* [3] | Rio Sarapiquí (Chirripó) | San Juan (1) | 13 | PG713 | SEG | 13 | 10.5246 | -84.0313 | Costa Rica | — | 21 | 6 | 0.872 | 0.054 | 0.0066 | 6.767 | 13 |
| *P. gillii* [3] | Rio Isla Grande (Chirripó) | San Juan (1) | 14 | PG636 | SEG | 1 | 10.3930 | -83.9682 | Costa Rica | — | NA | NA | NA | NA | NA | NA | 1 |
| *P. gillii* [3] | Rio Tortuguero (Tortuguero) | Tortuguero (2) | 16 | PG712 | SEG | 15 | 10.2594 | -83.8122 | Costa Rica | — | 18 | 5 | 0.705 | 0.088 | 0.0037 | 5.536 | 15 |
| *P. gillii* [3] | Unnamed tributary | Parismina (2) | 17 | PG710 | SEG | 7 | 10.1977 | -83.5687 | Costa Rica | — | 0 | 1 | 0.000 | 0.000 | 0.0000 | 0.000 | 7 |
| *P. gillii* [3] | Rio Herediana | Parismina (2) | 18 | PG703 | SEG | 5 | 10.1242 | -83.5562 | Costa Rica | — | 54 | 3 | 0.800 | 0.164 | 0.0214 | 25.920 | 5 |
| *P. gillii* [3] | Rio Reventazon | Parismina (2) | — | PG701 | SEG | 5 | 9.8723 | -83.6332 | Costa Rica | — | 18 | 2 | 0.400 | 0.237 | 0.0063 | 8.640 | 5 |
| *P. gillii* [3] | Rio Toro | Matina (2) | — | PG708 | SEG | 8 | 10.0168 | -83.2102 | Costa Rica | — | 46 | 3 | 0.679 | 0.122 | 0.0106 | 17.741 | 8 |
| *P. gillii* [3] | Unnamed lagoon | Matina (2) | — | PG707 | SEG | 8 | 9.8926 | -82.9723 | Costa Rica | — | 2 | 3 | 0.464 | 0.200 | 0.0004 | 0.771 | 8 |
| *P. gillii* [3] | Rio Carbon | Sixaola (2) | 20 | PG706 | SEG | 8 | 9.6231 | -82.8552 | Costa Rica | — | 12 | 3 | 0.607 | 0.164 | 0.0054 | 4.628 | 8 |
| *P. gillii* [3] | Rio Sixaola | Sixaola (2) | — | PG704 | SEG | 8 | 9.6320 | -82.8192 | Costa Rica | — | 0 | 1 | 0.000 | 0.000 | 0.0000 | 0.000 | 8 |
| **Means:** |  |  |  |  |  | **7.526** |  |  |  |  | **12.611** | **3** | **0.498** | **0.139** | **0.0045** | **5.586** | **7.526** |
|  |  |  |  |  |  |  |  |  |  |  |  |  |  |  |  |  |  |
| ***Xenophallus umbratilis*** |  |  |  |  |  | **131** |  |  |  |  |  |  |  |  |  |  |  |
| *X. umbratilis* [4] | Rio Zapote (Zapote) | San Juan (1) | — | Xu0625 | NWG | 8 | 10.7242 | -85.0664 | Costa Rica | — | 1 | 2 | 0.429 | 0.169 | 0.00038 | 0.386 | 8 |
| *X. umbratilis* [4] | Rio Bijagua (Zapote) | San Juan (1) | — | Xu0620 | NWG | 8 | 10.7277 | -84.0313 | Costa Rica | — | 3 | 3 | 0.464 | 0.200 | 0.00066 | 1.157 | 8 |
| *X. umbratilis* [4] | Unnamed tributary (Zapote) | San Juan (1) | — | Xu0621 | NWG | 8 | 10.7314 | -85.0553 | Costa Rica | — | 1 | 2 | 0.429 | 0.169 | 0.00038 | 0.386 | 8 |
| *X. umbratilis* [4] | Rio Venado (Frio) | San Juan (1) | 7 | Xu0719 | LSJTORG | 4 | 10.6448 | -84.8222 | Costa Rica | — | 1 | 2 | 0.500 | 0.265 | 0.00044 | 0.545 | 4 |
| *X. umbratilis* [4] | Rio Chimurria (Pocosol) | San Juan (1) | 9 | Xu0635 | LSJTORG | 8 | 10.7274 | -84.5582 | Costa Rica | — | 1 | 2 | 0.250 | 0.180 | 0.00022 | 0.386 | 8 |
| *X. umbratilis* [4] | Rio Sarapiquí (Chirripó) | San Juan (1) | 13 | Xu0713 | LSJTORG | 1 | 10.5245 | -84.0313 | Costa Rica | — | NA | NA | NA | NA | NA | NA | 1 |
| *X. umbratilis* [4] | Rio Isla Grande (Chirripó) | San Juan (1) | 14 | Xu0636 | LSJTORG | 8 | 10.3930 | -83.9682 | Costa Rica | — | 5 | 4 | 0.643 | 0.184 | 0.00110 | 1.928 | 8 |
| *X. umbratilis* [4] | Rio Corinto (Chirripó) | San Juan (1) | 15 | Xu0637 | COG | 8 | 10.2119 | -83.8865 | Costa Rica | — | 0 | 1 | 0.000 | 0.000 | 0.00000 | 0.000 | 8 |
| *X. umbratilis* [4] | Upper Rio Tortuguero | Tortuguero (2) | 16 | Xu0712 | LSJTORG | 8 | 10.2594 | -83.8122 | Costa Rica | — | 2 | 2 | 0.536 | 0.123 | 0.00094 | 0.771 | 8 |
| *X. umbratilis* [4] | Upper Rio Tortuguero | Tortuguero (2) | — | XuCar | LSJTORG | 1 | 10.3553 | -83.7375 | Costa Rica | — | NA | NA | NA | NA | NA | NA | 1 |
| *X. umbratilis* [4] | Unknown tributary | Parismina (2) | — | XuJimez | PAG | 2 | 10.2894 | -83.6100 | Costa Rica | — | 1 | 2 | 1.000 | 0.500 | 0.00088 | 1.000 | 2 |
| *X. umbratilis* [4] | Unnamed tributary | Parismina (2) | — | Xu0710 | PAG | 6 | 10.1978 | -83.6519 | Costa Rica | — | 1 | 2 | 0.533 | 0.172 | 0.00047 | 0.438 | 6 |
| *X. umbratilis* [4] | Quebrada Piecueca (San Carlos) | San Juan (1) | 10 | XuTigra | UG | 2 | 10.3517 | -84.5881 | Costa Rica | — | 0 | 1 | 0.000 | 0.000 | 0.00000 | 0.000 | 2 |
| *X. umbratilis* [4] | Lake Arenal (San Carlos) | San Juan (1) | — | Xu0631 | UG | 8 | 10.5486 | -84.9808 | Costa Rica | — | 1 | 2 | 0.250 | 0.180 | 0.00022 | 0.386 | 8 |
| *X. umbratilis* [4] | Lake Arenal (San Carlos) | San Juan (1) | — | Xu0607 | UG | 8 | 10.5597 | -84.9697 | Costa Rica | — | 3 | 4 | 0.750 | 0.139 | 0.00081 | 1.157 | 8 |
| *X. umbratilis* [4] | Lake Arenal (San Carlos) | San Juan (1) | — | Xu0632 | UG | 8 | 10.5603 | -84.9403 | Costa Rica | — | 1 | 2 | 0.536 | 0.123 | 0.00047 | 0.386 | 8 |
| *X. umbratilis* [4] | Lake Arenal (San Carlos) | San Juan (1) | — | Xu0633 | UG | 4 | 10.5064 | -84.8458 | Costa Rica | — | 0 | 1 | 0.000 | 0.000 | 0.00000 | 0.000 | 4 |
| *X. umbratilis* [4] | Lake Arenal (San Carlos) | San Juan (1) | — | Xu0604 | UG | 3 | 10.5014 | -84.8406 | Costa Rica | — | 0 | 1 | 0.000 | 0.000 | 0.00000 | 0.000 | 3 |
| *X. umbratilis* [4] | Lake Arenal (San Carlos) | San Juan (1) | — | Xu9821 | UG | 8 | 10.4922 | -84.8358 | Costa Rica | — | 2 | 3 | 0.464 | 0.200 | 0.00044 | 0.771 | 8 |
| *X. umbratilis* [4] | Unknown tributary (San Carlos) | San Juan (1) | — | Xu9829 | UG | 8 | NA | NA | Costa Rica | — | 1 | 2 | 0.250 | 0.180 | 0.00022 | 0.386 | 8 |
| *X. umbratilis* [4] | Lake Arenal (San Carlos) | San Juan (1) | — | Xu0634 | UG | 4 | 10.4736 | -84.8222 | Costa Rica | — | 0 | 1 | 0.000 | 0.000 | 0.00000 | 0.000 | 4 |
| *X. umbratilis* [4] | Rio Agua Caliente (San Carlos) | San Juan (1) | — | Xu0717 | UG | 4 | 10.4350 | -84.7233 | Costa Rica | — | 1 | 2 | 0.500 | 0.265 | 0.00044 | 5.450 | 4 |
| *X. umbratilis* [4] | La Vuelta del Borracho (San Carlos) | San Juan (1) | — | Xu0718 | UG | 4 | 10.4275 | -84.7522 | Costa Rica | — | 0 | 1 | 0.000 | 0.000 | 0.00000 | 0.000 | 4 |
| **Means:** |  |  |  |  |  | **5.696** |  |  |  |  | **1.190** | **2** | **0.359** | **0.145** | **0.00038** | **0.740** | **5.696** |
| **Overall minimum value:** |  |  |  |  |  |  |  |  |  |  | **0** | **1** | **0** | **0** | **0** | **0** | **1** |
| **Overall maximum value:** |  |  |  |  |  |  |  |  |  |  | **54** | **7** | **1** | **0.5** | **0.0244** | **25.92** | **54** |

This table presents detailed sampling information for all three species in our study, including site names (with bodies of water that each sampled tributary flows into given in parentheses) where local sub-populations were sampled, site IDs corresponding to map numbers in **Fig. 2**, population group membership as inferred from BARRIER analyses (**Fig. 3**), number of samples (*N*), and latitude and longitude data in decimal degrees. For each site, we also list GenBank accession numbers (nos.) corresponding to sequences generated and/or analyzed for all individuals from that site. Numbers of segregating sites (*S*) determining the number of haplotypes (*h*), haplotype diversity (*Hd*) and its standard error (s.e.), nucleotide diversity (π), Watterson's theta (*θ_w_*, an estimator of population mutation rate), and *N* used for summary statistics calculations are presented. At the bottom of each species list, intraspecific mean values are given in bold for *N* and summary statistics (see text for further details). The overall ranges (min. and max. across sites within species, across taxa) of each statistic are summarized at the bottom of the table in bold. Symbols and abbreviations: §, indicates some samples were removed prior to analyses for a given site (e.g., due to missing data) and this explains discrepancies between overall *N*s (column 7) and sample sizes summary statistics were calculated from; dr., drainage(s); NA, not available.

**References**

1. Hrbek T, Seckinger J, Meyer A (2007) A phylogenetic and biogeographic perspective on the evolution of poeciliid fishes. Mol Phylogenet Evol 43: 986-998.
2. Doadrio I, Perea S, Alcaraz L, Hernandez N (2009) Molecular phylogeny and biogeography of the Cuban genus *Girardinus* Poey, 1854 and relationships within the tribe Girardinini (Actinopterygii, Poeciliidae). Mol Phylogenet Evol 50: 16-30.
3. Lee JB, Johnson JB (2009) Biogeography of the livebearing fish *Poecilia gillii* in Costa Rica: are phylogeographical breaks congruent with community boundaries? Mol Ecol 18: 4088-4101.
4. Jones CP, Johnson JB (2009) Phylogeography of the livebearer *Xenophallus umbratilis* (Teleostei: Poeciliidae): glacial cycles and sea level change predict diversification of a freshwater tropical fish. Mol Ecol 18: 1640-1653.
