# Supplementary material for: Testing for shared biogeographic history in the lower Central American freshwater fish assemblage using comparative phylogeography: concerted, independent, or multiple evolutionary responses?
Source: Ecol Evol. 2014 Apr 10;4(9):1686–705. doi: 10.1002/ece3.1058 (PMC4063468; doi:10.1002/ece3.1058)
Supplement: Supplementary file 7 [file ece30004-1686-SD7.docx]

**Table S2 DNA substitution models selected using DT-ModSel**

| Species | DNA dataset | *N* | bp | Best model | Analysis |
| --- | --- | --- | --- | --- | --- |
| ***Alfaro cultratus*** | |  |  |  |  |
|  | Full cyt*b* database | 355 | 601 | HKY+*I* | beast (BSP), DnaSP, tcs (network) |
|  | 1^st^ codon pos. | 355 | 201 | K80 | beast (BSP) |
|  | 2^nd^ codon pos. | 355 | 200 | F81 | beast (BSP) |
|  | 3^rd^ codon pos. | 355 | 200 | TrN+Γ | beast (BSP) |
|  | *A. cultratus* cyt*b* haplotypes (*N* = 46) + 1 *A. huberi* outgroup sequence | 47 | 601 | HKY+Γ | garli (ML) |
|  | 1^st^ codon pos. | 47 | 201 | TrNef | garli (ML) |
|  | 2^nd^ codon pos. | 47 | 200 | F81 | garli (ML) |
|  | 3^rd^ codon pos. | 47 | 200 | HKY+Γ | garli (ML) |
| ***Poecilia gillii*** | |  |  |  |  |
|  | Full cyt*b* database | 143 | 1140 | TrN+*I* | beast (BSP), DnaSP, tcs (network) |
|  | 1^st^ codon pos. | 143 | 379 | K80 | beast (BSP) |
|  | 2^nd^ codon pos. | 143 | 379 | F81 | beast (BSP) |
|  | 3^rd^ codon pos. | 143 | 379 | TrN+Γ | beast (BSP) |
|  | *P. gillii* cyt*b* haplotypes (*N* = 37) + 1 *P. mexicana* outgroup sequence | 38 | 1140 | TrN+*I* | garli (ML) |
|  | 1^st^ codon pos. | 38 | 379 | K80 | garli (ML) |
|  | 2^nd^ codon pos. | 38 | 379 | F81 | garli (ML) |
|  | 3^rd^ codon pos. | 38 | 379 | TrN+*I* | garli (ML) |
| ***Xenophallus umbratilis*** | |  |  |  |  |
|  | Full cyt*b* database | 131 | 1140 | TrN+Γ | beast (BSP), DnaSP, tcs (network) |
|  | 1^st^ codon pos. | 131 | 379 | K80 | beast (BSP) |
|  | 2^nd^ codon pos. | 131 | 379 | F81 | beast (BSP) |
|  | 3^rd^ codon pos. | 131 | 379 | TrN | beast (BSP) |
|  | *Xenophallus* cyt*b* haplotypes (*N* = 36) + 1 *Priapichthys annectens* outgroup sequence | 37 | 1140 | HKY+Γ | garli (ML) |
|  | 1^st^ codon pos. | 37 | 379 | SYM | garli (ML) |
|  | 2^nd^ codon pos. | 37 | 379 | HKY | garli (ML) |
|  | 3^rd^ codon pos. | 37 | 379 | TrN | garli (ML) |

**Model selection analyses using the decision theory algorithm in DT-ModSel [1] supported different best-fit models of DNA evolution for different datasets across taxa, including datasets filtered by codon positions. This table lists model selection results for intraspecific cyt*b* datasets analyzed in this study, as well as the analyses that each dataset (thus molecular model) was used in. Symbols and abbreviations: *Γ*, gamma-distributed rate variation; bp, number of nucleotide base pairs; BSP, Bayesian skyline plot and associated demographic modeling and Bayes factor analyses; DnaSP, DNA polymorphism, mismatch distribution, and neutrality statistics analyses conducted in the program by the same name; *I*, parameter representing proportion of invariable sites; ML, phylogenetic maximum likelihood analyses estimating haplotype gene trees; *N*, sample size. Although different model selection algorithms, such as ModelTest [2] and jModelTest [3], are available that have historically been more widely used than DT-ModSel, we preferred to use this software for our substitution model selection analyses because DT-ModSel has been shown to recover better models than these other programs [1].**

**References**

1. Minin V, Abdo Z, Joyce P, Sullivan J (2003) Performance-based selection of likelihood models for phylogeny estimation. Syst Biol 52: 674-683.
2. Posada D, Crandall KA (1998) MODELTEST: testing the model of DNA substitution. Bioinformatics 14: 817-818.
3. Posada D (2008) jModelTest: phylogenetic model averaging. Mol Biol Evol 25: 1253-1256.
