# Supplementary material for: Testing for shared biogeographic history in the lower Central American freshwater fish assemblage using comparative phylogeography: concerted, independent, or multiple evolutionary responses?
Source: Ecol Evol. 2014 Apr 10;4(9):1686–705. doi: 10.1002/ece3.1058 (PMC4063468; doi:10.1002/ece3.1058)
Supplement: Supplementary file 8 [file ece30004-1686-SD8.docx]

**Table S3 Cyt*b* DNA polymorphism levels within and among drainage basins**

| Species | Drainage | *S* | *h* | *Hd* | s.e. | π | *k* | *θ_w_* | Mean *N* |
| --- | --- | --- | --- | --- | --- | --- | --- | --- | --- |
| ***A. cultratus*** |  |  |  |  |  |  |  |  |  |
|  | **San Juan** | 14.154 | 3.692 | 0.449 | 0.097 | 0.005 | 3.242 | 4.110 | 22.250 |
|  | **Tortuguero** | 27.000 | 6.000 | 0.818 | 0.084 | 0.016 | 9.758 | 8.941 | 12.000 |
|  | **Parismina** | 7.500 | 4.000 | 0.596 | 0.106 | 0.004 | 2.492 | 2.261 | 16.000 |
|  | **Sixaola** | 27.500 | 5.000 | 0.927 | 0.158 | 0.021 | 12.676 | 12.055 | 11.000 |
|  | **mean:** | **19.038** | **4.673** | **0.698** | **0.111** | **0.012** | **7.042** | **6.842** | **15.313** |
| ***P. gillii*** |  |  |  |  |  |  |  |  |  |
|  | **San Juan** | 7.700 | 3.000 | 0.530 | 0.153 | 0.0033 | 3.795 | 3.732 | 7.182 |
|  | **Tortuguero** | 18.000 | 5.000 | 0.705 | 0.088 | 0.0037 | 4.210 | 5.536 | 15.000 |
|  | **Parismina** | 24.000 | 2.000 | 0.400 | 0.134 | 0.0092 | 10.533 | 11.520 | 5.667 |
|  | **Matina** | 24.000 | 3.000 | 0.572 | 0.161 | 0.0055 | 6.286 | 9.256 | 8.000 |
|  | **Sixaola** | 6.000 | 2.000 | 0.304 | 0.082 | 0.0027 | 3.072 | 2.314 | 8.000 |
|  | **mean:** | **15.940** | **3.000** | **0.502** | **0.124** | **0.0049** | **5.579** | **6.472** | **8.770** |
| ***Xenophallus*** |  |  |  |  |  |  |  |  |  |
|  | **San Juan** | 1.176 | 2.000 | 0.307 | 0.122 | 0.00033 | 0.372 | 0.761 | 5.889 |
|  | **Tortuguero** | 2.000 | 2.000 | 0.536 | 0.123 | 0.00094 | 1.071 | 0.771 | 4.500 |
|  | **Parismina** | 1.000 | 2.000 | 0.767 | 0.336 | 0.00068 | 0.767 | 0.719 | 4.000 |
|  | **mean:** | **1.392** | **2.000** | **0.536** | **0.194** | **0.00065** | **0.736** | **0.750** | **4.796** |

**Refer to text for description of DNA polymorphism statistics. *Xenophallus* samples from Rio Tempisque, while included in our other analyses for this species (e.g., of the full cyt*b* dataset; see Table S1), are excluded here because this drainage does not occur in the main study area (thus, data from this drainage are excluded from mean back-arc drainage group comparisons in the text).**
