# Supplementary material for: Testing for shared biogeographic history in the lower Central American freshwater fish assemblage using comparative phylogeography: concerted, independent, or multiple evolutionary responses?
Source: Ecol Evol. 2014 Apr 10;4(9):1686–705. doi: 10.1002/ece3.1058 (PMC4063468; doi:10.1002/ece3.1058)
Supplement: Supplementary file 9 [file ece30004-1686-SD9.docx]

**Table S4. Model priors, estimated number and timing of divergence events, and Bayes factors from MTML-msBayes.** Results are presented for four coalescent models (M1-M4) run in MTML-msBayes. Bayes factors were used to conduct hypotheses tests of posterior support for simultaneous divergence (e.g., *Ψ=1*) versus other hypotheses.

| **Model:** | **M1** | **M2** | **M3** | **M4** | **mean:** |
| --- | --- | --- | --- | --- | --- |
| **Prior settings** |  | |  |  |  |
| **upper *θ*** | 0.0049 | 0.01 | 0.05 | 0.0049 | – |
| **lower *θ*** | 4 × 10^-8^ | 4 × 10^-8^ | 4 × 10^-8^ | 4 × 10^-8^ | – |
| **upper τ** | 2 | 2 | 2 | 2 | – |
| **no. τ classes (*Ψ*)** | ^1^0 | 0 | 0 | 0 | – |
| ***Nm*** | 0 | 0 | 0 | 0 | – |
| **up. ancestral *θ*** | 0.5 | 0.5 | 0.5 | 0.25 | – |
| **constrain=** | 0 | 0 | 0 | 0 | – |
|  |  |  |  |  |  |
| **Posterior estimates** | | |  |  |  |
| **mean *Ψ* ^2^** | 2.582 | 2.291 | 2.132 | 2.157 | 2.219 |
| **BPP *Ψ*=1** | 0.0859 | 0.149 | 0.218 | 0.203 | 0.211 |
| **mode *E*[τ]** | 0.644 | 1.173 | 0.395 | 1.0497 | 0.944 |
| ***E*[τ] 95% Cis** | [0.000,1.312] | [0.445,1.527] | [0.0208,0.585] | [0.270,1.652] | – |
| **Div. time (Ma) ^3^** | 0.158 | 0.587 | 0.988 | 0.257 | 0.543 |
| **Div. time 95% CIs** | [0.000,0.321] | [0.223,0.764] | [0.0520,1.463] | [0.0662,0.405] | – |
| **Div. time (Ma) ^4^** | 0.351 | 1.303 | 2.194 | 0.572 | 1.208 |
| **Div. time 95% CIs** | [0.000,0.714] | [0.494,1.697] | [0.116,3.250] | [0.147,0.899] | – |
| **mean *Ω*** | 0.626 | 0.269 | 0.312 | 0.481 | 0.367 |
| ***Ω* 95% HPDs** | [0.252,1.218] | [0.000,0.657] | [0.000,0.734] | [0.104,1.0001] | – |
|  |  |  |  |  |  |
| **Bayes factors (*B*_10_)** | | |  |  |  |
| **Comparison** |  | |  |  |  |
| ***Ψ*=1 vs. *Ψ*>1** | 0.9955 | 0.9296 | 0.7634 | 1.0181 | 1.0175 |
| ***Ψ*>1 vs. *Ψ*=1** | **4.018** | **4.3028** | **5.2391** | **3.9287** | **4.0771** |
| ***Ω*>0.01 vs. *Ω*<0.01** | 1.535 | 1.525 | 1.539 | **1.558** | 1.539 |
| ***Ψ*=3 vs. *Ψ*<3** | 0.9643 | 0.9469 | 1.0833 | 1.009 | 0.960 |

**The population mutation parameter *θ* is in units of per site per generation. In the mean divergence time hyper-parameter *E*[τ], τ is the mean divergence time of the population pairs (calculated from *τ_1_, …, τ_Y_* population pairs), in coalescent time units of 4*N* generations. In the *Nm* parameter representing the effective number of migrants per generation, *m* denotes the probability of symmetric post-divergence migration between sister lineages. Results given in this table are based on coalescent simulations (5 × 10^6^ iterations) of *Y*=3 population pairs, following which an accept/reject algorithm with tolerance set to 0.0002 was used to create a distribution of 999 draws from the prior, to approximate the joint posterior distribution. The only exceptions are the *Ω* Bayes factor comparisons (*B*_10_), which were based on 9,999 draws from the prior. Estimated 95% confidence intervals are given in brackets. *B*_10_ Bayes factors >2 are presented in bold, and *B*_10_ values indicating ‘substantial’ support in favor of the alternative hypothesis (values >3.2; based on guidelines in Jeffreys [1]) are further underlined. Abbreviations: BPP, Bayesian posterior probability; CIs, confidence intervals; Div. time, divergence time; Ma, millions of years ago (assuming generation time = 1 yr/generation); no., number of; up., upper.**

**^1^Here, zero specifies that *Ψ* were drawn from a set of 1-3, or up to the total number of taxon pairs.**

**^2^Posterior probability estimated from polychotomous regression (i.e., local multinomial logit regression).**

**^3^Calculated as Div. time = *E*[τ] × [(0.5 × upper *θ*)/μ], assuming the standard pairwise 2% vertebrate mtDNA rate [2,3].**

**^4^Similar to ^3^, but calculated assuming the pairwise 0.9% salmonid mtDNA rate [4].**

**References**

1. Jeffreys H (1961) Theory of probability. Third Edition. Oxford, UK: Oxford University Press. 447 p.
2. Brown WM, George M Jr, Wilson AC (1979) Rapid evolution of animal mitochondrial DNA. Proc Natl Acad Sci USA 76: 1967-1971.
3. Wilson AC, Cann RL, Carr SM, George M, Gyllensten UB, Helm KM, Bychowski R, Higuchi RG, Palumbi SR, Prager EM, Sage RD, Stoneking M (1985) Mitochondrial DNA and two perspectives on evolutionary genetics. Biol J Linn Soc 26: 385-400.
4. Martin AP, Palumbi SR (1993) Body size, metabolic rate, generation time, and the molecular clock. Proc Natl Acad Sci USA 90: 4087-4091.
