# Supplementary material for: Testing for shared biogeographic history in the lower Central American freshwater fish assemblage using comparative phylogeography: concerted, independent, or multiple evolutionary responses?
Source: Ecol Evol. 2014 Apr 10;4(9):1686–705. doi: 10.1002/ece3.1058 (PMC4063468; doi:10.1002/ece3.1058)
Supplement: Supplementary file 10 [file ece30004-1686-SD10.docx]

**Appendix S1: Sampling and outgroups details**

In addition to sampling information provided in the main text and figures, we provide detailed information on collection localities for each of our focal species in **Table S1** of the Supporting Information, including site names and geographical coordinates. Geographical coordinates are given in decimal degrees format (these were used during samova and barrier analyses; see **Appendix S2**) and should be highly accurate as we took them from the ground in Costa Rica using hand-held GPS devices (Honduran samples were similarly derived on-site by W. Matamoros, who provide the samples). **Table S1** also lists GenBank accession numbers for new *Alfaro cultratus* sequences generated in this study, as well as those for sequences of *Poecilia gillii* (from Lee and Johnson [1]) and *Xenophallus umbratilis* (from Jones and Johnson [2]) used in this study.

As noted in the main text, we used one or more *Alfaro huberi* samples collected for this study as outgroups during our *A. cultratus* analyses. For maximum likelihood (ML) phylogenetic analysis in garli, we used one *A. huberi* sample (GenBank accession no.: XXXXXXX, haplotype 47, **Table S1**), while for the Hudson-Kreitman-Aguadé test (HKA; [3]) conducted in DnaSP, we used as data for the outgroup species all seven *A. huberi* samples listed in **Table S1**, which collapsed into three distinct cyt*b* haplotypes (H47-H49). Outgroups added to the other species cyt*b* alignments during garli analyses included published cyt*b* sequences for two additional species of livebearing fishes (Poeciliidae) obtained from GenBank. Specifically, based on phylogenetic hypotheses of Hrbek *et al.* [4] and Ptacek and Breden [5], *Priapichthys annectens* (GenBank no: EF017542, genotype/isolate ID “Panne” from Hrbek *et al.* [4]) was the outgroup for *Xenophallus*, and *Poecilia mexicana* (GenBank no: FJ178776, genotype/isolate ID “3211MEX”, locality “Col River, Veracruz” Mexico, from Doadrio *et al.* [6]) was the outgroup for *P. gillii*. These same sequences served as outgroups during HKA tests conducted on the full cyt*b* databases of *P. gillii* and *Xenophallus* used in this study.

**References**

1. Lee JB, Johnson JB (2009) Biogeography of the livebearing fish *Poecilia gillii* in Costa Rica: are phylogeographical breaks congruent with community boundaries? Mol Ecol 18: 4088-4101.
2. Jones CP, Johnson JB (2009) Phylogeography of the livebearer *Xenophallus umbratilis* (Teleostei: Poeciliidae): glacial cycles and sea level change predict diversification of a freshwater tropical fish. Mol Ecol 18: 1640–1653.
3. Hudson RR, Kreitman M, Aguadé M (1987) A test of neutral molecular evolution based on nucleotide data. Genetics 116: 153-159.
4. Hrbek T, Seckinger J, Meyer A (2007) A phylogenetic and biogeographic perspective on the evolution of poeciliid fishes. Mol Phylogenet Evol 43: 986-998.
5. Ptacek MB, Breden F (1998) Phylogenetic relationships among the mollies (Poeciliidae: *Poecilia: Mollienesia* group) based on mitochondrial DNA sequences. J Fish Biol 53: 64-81.
6. Doadrio I, Perea S, Alcaraz L, Hernandez N (2009) Molecular phylogeny and biogeography of the Cuban genus *Girardinus* Poey, 1854 and relationships within the tribe Girardinini (Actinopterygii, Poeciliidae). Mol Phylogenet Evol 50: 16-30.
