# Supplementary material for: Testing for shared biogeographic history in the lower Central American freshwater fish assemblage using comparative phylogeography: concerted, independent, or multiple evolutionary responses?
Source: Ecol Evol. 2014 Apr 10;4(9):1686–705. doi: 10.1002/ece3.1058 (PMC4063468; doi:10.1002/ece3.1058)
Supplement: Supplementary file 11 [file ece30004-1686-SD11.docx]

**Appendix S2: samova and barrier methods and results**

The samova algorithm [1] and Monmonier’s [2] algorithm, as implemented for studying phylogeographic data in barrier [3,4], comprise two recent and widely used methods for detecting the presence of genetic barriers and population structure. Several previous single species phylogeography studies have used barrier and samova to identify groups of populations, which were then used for further statistical population genetics analyses of the same datasets (e.g., to conform to the expectation of the methods/models that there is no underlying population structure in the data influencing the results), and to estimate areas where important landscape features or environmental changes may have historically isolated local populations or impeded gene flow (e.g., [5]). It is also clear from the literature that these methods are well suited for comparative analyses. In-line with our study, comparative phylogeographical analyses such as a well-known review and meta-analysis of eastern North American phylogeography by Soltis *et al.* [6], and a recent analysis by Poelchau and Hamrick [7] of three codistributed lower Central American tree species that today share overlapping distributions relative to our study taxa, have used Monmonier’s algorithm to identify important genetic barriers within multiple codistributed taxa, in order to test for spatial phylogeographical congruence.

Genetic ‘barriers’ are areas of maximum rates of genetic change across a landscape (discussed in [1]), and while both samova and barrier use Voroni network-based methods for defining genetic barriers, these methods are different and therefore highly complementary. For example, because samova directly estimates population structure (positions of homogeneous, maximally genetically differentiated groups or “populations”) while taking spatial sampling positions into account and indirectly defining genetic barriers as areas between the inferred populations, whereas Monmonier’s algorithm directly reconstructs genetic barriers and thus indirectly identifies population grouping schemes [1]. In either case, both of these methods permit recovering an estimate of the spatial positions of the unknown number, *K*, of actual (presumably panmictic) homogeneous breeding populations within a species. Dupanloup *et al.* [1] showed, through population genetics simulations, that samova performs best out of the two methods at identifying maximally genetically diverged groups, whereas barrier is more proficient at finding the actual number of *K* population groupings.

We implemented both of these methods as a combined test of spatial-genetic congruence among our three focal freshwater fish taxa, to evaluate whether these species exhibited shared patterns of genetic barriers reflecting potentially shared evolutionary history. We used each method because it is not firmly established which method is best for identifying comparative phylogeographical congruence. Thus we preferred to look for cross-validation across methods as evidence that our comparative inferences were ‘strongly supported’, i.e., repeatable and robust to different underlying assumptions of different methods. Here, it is important to note, as pointed out by Garrick *et al.* [8], that seeking cross-validation in this way is only valid when results are compared across methods that have similar underlying purposes, as in our study. In our study, comparative phylogeographical congruence would be strongly supported by similar geographical positions of inferred population groups and barriers across all three species. However, we assumed that rigid spatial congruence (of inferred barriers/populations) across taxa along all network edges was not a requirement for arriving at a basis for biologically meaningful interpretation of the data. Instead, we recognized that identifying partial spatial-genetic congruence in a limited part of the study area would still present an opportunity for making further inferences, if only over smaller spatial scales than the entire sampling extent.

Population genetic simulations suggest the largest mean *F_CT_* value among a series of samova models with different initial settings may accurately recover the unknown number of groups (*K*), and that the point at which increasing *F_CT_* values asymptote often represents a meaningful estimate of *K* [1]. Our rationale behind interpreting the ‘best’ number of groups determined from our samova model results stemmed explicitly from these findings. However, we used Φ*_CT_*, the *F_CT_* analog for DNA sequences analyzed under the analysis of variance framework [9], as the basis of our interpretations. Both of these “*_CT_*”-subscripted statistics represent the amount of molecular genetic variance present in the overall sample that is explained by among-group variation. It is also noteworthy to point out that *Xenophallus* and *P. gillii* samova results conformed to the expectation that Φ*_CT_* increase with *K* [1], with Φ-value plateaus respectively supporting *K* = 9 and *K* = 6 distinct groups (**Fig. S2**). However, this behavior was not observed in *A. cultratus*. In light of the inferred patterns of phylogenetic clades of *A. cultratus* and their relationships based on maximum-likelihood phylogenetic gene tree analyses and network analyses, which corresponded to the inferred *K* = 2 samova groups (and thus also to the barriers inferred by running Monmonier’s algorithm on the *A. cultratus* data), it seemed highly appropriate to interpret this deviation in *A. cultratus* as a natural outcome of *K* = 2 being the best model. In other words, our interpretation in light of additional evidence is that *A. cultratus* likely deviates from the expectation that Φ*_CT_* increase with *K*, as a consequence of Φ*_CT_* peaking at *K =* 2 groups.

To round out our discussion of Φ-statistics above and in the main text, we note here that in contrast to Φ*_CT_*, Φ*_SC_* is the correlation of the diversity of random haplotypes within sub-populations (localities) relative to random pairs from the same group of sub-populations (within regions); whereas Φ*_ST_* is the correlation of random haplotypes within sub-populations relative to random pairs drawn from the entire dataset (analogous to *F*_ST_). In addition to Φ*_CT_*, we also report Φ*_SC_* and Φ*_ST_* from independent amovas testing what we determined to be the best grouping schemes inferred from our samova/barrier models (see Table 2).

**References**

1. Dupanloup I, Schneider S, Excoffier L (2002) A simulated annealing approach to define the genetic structure of populations. Mol Ecol 11: 2571-2581.
2. Monmonier MS (1973) Maximum-difference barriers: an alternative numerical regionalization method. Geogr Anal 3: 245-261.
3. Manni FE, Guerard E, Heyer E (2004a) Geographical patterns of (genetic, morphologic, linguistic) variation: how barriers can be detected by "Monmonier's algorithm". Hum Biol 76: 173-190.
4. Manni FE, Guerard E, Heyer E (2004b) BARRIER 2.2. Museum of Mankind, Paris, France. Available at: http://www.mnhn.fr/mnhn/ecoanthropologie/software/barrier.html.
5. Ribeiro RA, Lemos-Filho JP, Ramos ACS, Lovato MB (2011) Phylogeography of the endangered rosewood *Dalbergia nigra* (Fabaceae): insights into the evolutionary history and conservation of the Brazilian Atlantic Forest. Heredity 106: 46-57.
6. Soltis DE, Morris AB, McLachlan JS, Manos PS, Soltis PS (2006). Comparative phylogeography of unglaciated eastern North America. Mol Ecol 15: 4261-4293.
7. Poelchau MF, Hamrick JL (2011) Comparative phylogeography of three common Neotropical tree species. J Biogeogr, doi:10.1111/j.1365-2699.2011.02599.x.
8. Garrick RC, Caccone A, Sunnucks P (2010) Inference of population history by coupling exploratory and model-driven phylogeographic analyses. Int J Mol Sci 11: 1190-1227.
9. Excoffier L, Smouse PE, Quattro JM (1992) Analysis of molecular variance inferred from metric distances among DNA haplotypes: application to human mitochondrial DNA restriction data. Genetics 131: 479-491.
