# Supplementary material for: Testing for shared biogeographic history in the lower Central American freshwater fish assemblage using comparative phylogeography: concerted, independent, or multiple evolutionary responses?
Source: Ecol Evol. 2014 Apr 10;4(9):1686–705. doi: 10.1002/ece3.1058 (PMC4063468; doi:10.1002/ece3.1058)
Supplement: Supplementary file 12 [file ece30004-1686-SD12.docx]

**Appendix S3: Coalescent divergence time estimation: IMa2 methods**

The ‘isolation-with-migration’ model implemented through the MCMC procedure available in IMa2 uses a procedure that samples many coalescent genealogies, uses them to capture what the data says about the parameters in the model (*m*_1_, *m*_2_, *θ*_1_, *θ*_2_, *θ*_A_, *t*), and then uses the genealogies to estimate the posterior density of the parameters [1-3]. IMa2 also estimates the TMRCA from the many genealogies sampled. We ran IMa2 with simple two-population models, although the program accommodates >1 ancestral population thus >2 modern populations, because we analyzed a single locus, and these other more complicated models require much more data [4]. It is important to note that, despite providing reliable methods for modeling population history while accounting for potentially confounding processes (e.g., migration, mutational stochasticity), coalescent-genealogy sampling methods including IMa2 make several limiting assumptions and have their own peculiarities. Because space was not permitting in the main text we briefly discuss (i) the assumptions and limitations of this program here, and we also give more detailed information on our IMa2 (ii) analyses and (iii) results.

The first assumption that IMa2 makes is (1) that the data being analyzed are neutrally evolving DNA markers and not influenced by the effects of directional selection or purifying selection (e.g., selective sweeps) [2]. We tested this assumption and found that our mtDNA data met the expectation of neutrality, e.g., based on HKA tests (see “Genetic diversity and neutrality” section, Results). (2) IMa2 also assumes no recombination, and our data meet this criterion: mtDNA are not subject to detectable recombination events. (3) The model implemented in IMa2 also assumes that the populations are not exchanging migrants with any other populations than those modeled and that migration, and that gene flow occurs at a constant rate following population splitting events [2]. Several of the genetically meaningful population groups that we conducted IMa2 analyses on (see barrier results) are allopatrically distributed and bounded on their southwestern sides by the North American continental divide (Figs. 1-3); thus these groups seem to fit assumption 2 above, as the next proximal populations sampled seem sufficiently geographically close and isolated as to exclude the possibility of exchange with other (e.g., unsampled) populations. (4) IMa2 also (unlike its predecessor, IM) assumes constant population sizes following initial population splitting (assuming a two-population case or model). Given that our mismatch distribution and neutrality tests generally inferred a shared pattern of population size-constancy for each barrier-inferred population group that we modeled in IMa2, our data are also well suited for IMa2 analysis because they fit this assumption (see “Historical demographic congruence” section, in Results). Moreover, even at the species level, there was only strong evidence for past population dynamics (e.g., in Bayesian skyline models) in *P. gillii*, not the other species (Table 4).

Another relevant point to note is that IMa2 and similar programs cannot identify the timing of migration (whether it occurred before during or after population splitting, or only at present, etc.), although coalescent-inferred migration events most likely occur (or are observed) near the present [5]. As a result, we did not attempt to infer, or test hypotheses based on, posterior-derived estimates of migration timing, although developing methods to address such questions would be a worthwhile endeavor for future research.

In terms of settings, our IMa2 runs employed Hasegawa-Kishino-Yano (HKY) substitution models [6,7]. We used this model because it is the most appropriate model implemented in IMa2 for DNA sequence data, allowing for multiple substitutions and different transition and transversion rates. In contrast, other models selected for the data by DT-ModSel for our population groups (data not shown) are not implemented in the IMa2 program. Using burn-in periods of 10^6^ steps followed by 3 × 10^6^ post-burn-in steps yielded reliable estimates of most parameters in most cases, based on sufficient convergence (e.g., stable trendline plots) and swapping rates of chains (e.g., splitting times were updated at higher rates in higher numbered chains, suggesting acceptable update rates).

In terms of results, our finding that the posterior distributions of *t* values (and estimates of other parameters, but usually only when *m* was not set equal to zero) often peaked at relatively lower *t* values, dropped, and then converged to approximately constant non-zero values is unremarkable. This pattern in the posterior is a common result of single-locus analyses that, despite being non-optimal, still allows excluding the equilibrium migration hypothesis in many cases [8], including our study. In other words, this pattern in our data indicates the peak likelihood represents a model with diverged populations, and this model was more likely than infinite, equilibrium migration, allowing us to exclude this latter hypothesis. Space was also prohibiting in the main text to permit some discussion of other results. For example, whereas we estimated non-zero *m* in *A. cultratus*, peak posterior m values or HPD ranges indicated that on-going gene flow was effectively zero in *P. gillii* and *Xenophallus*. Here, a practical point of note is that, in such cases, uniform *m* priors (the default) are ‘truly’ non-informative in IMa2. Thus, for ‘zero-migration’ population pairs found in *P. gillii* and *Xenophallus*, we conducted additional runs specifying *m* = 0 and these allowed us to achieve better convergence and *θ* and *t* parameter estimates, which we report. To account for this issue while permitting low levels of migration, JCB re-ran the IMa2 models for these zero-migration pairs under exponential *m* priors (-j7 option), modeling migration as a decreasing function with a peak at zero. Results of these exponential-migration runs did not substantially alter or depart from results inferred in the other runs (unpublished data).

**References**

1. Hey J, Nielsen R (2004) Multilocus methods for estimating population sizes, migration rates and divergence time, with applications to the divergence of *Drosophila pseudoobscura* and *D. persimilis*. Genetics 167: 747-760.
2. Hey J, Nielsen R (2007) Integration within the Felsenstein equation for improved Markov chain Monte Carlo methods in population genetics. Proc Natl Acad Sci USA 104: 2785-2790.
3. Hey J (2010) Isolation with migration models for more than two populations. Mol Biol Evol 27: 905-920.
4. Pinho C, Hey J (2010) Divergence with gene flow: models and data. Annu Rev Ecol Evol S 41: 215-230.
5. Sousa VC, Grelaud A, Hey J (2011) On the nonidentifiability of migration time estimates in isolation with migration models. Mol Ecol 20: 3956-3962.
6. Hasegawa M, Kishino H, Yano T (1985) Dating of the human-ape splitting by a molecular clock of mitochondrial DNA. J Mol Evol 22: 160-174.
7. Palsbøll PJ, Berube M, Aguilar A, Notarbartolo di Sciara G, Nielsen R (2004) Discerning between recurrent gene flow and recent divergence under a finite-site mutation model applied to North Atlantic and Mediterranean sea fin whale (*Balaenoptera physalus*) populations. Evolution 58: 670-675.
8. Nielsen R, Beaumont MA (2009) Statistical inferences in phylogeography. Mol Ecol 18: 1034-1047.
