# Supplementary material for: Testing for shared biogeographic history in the lower Central American freshwater fish assemblage using comparative phylogeography: concerted, independent, or multiple evolutionary responses?
Source: Ecol Evol. 2014 Apr 10;4(9):1686–705. doi: 10.1002/ece3.1058 (PMC4063468; doi:10.1002/ece3.1058)
Supplement: Supplementary file 1 [file ece30004-1686-SD1.pdf]

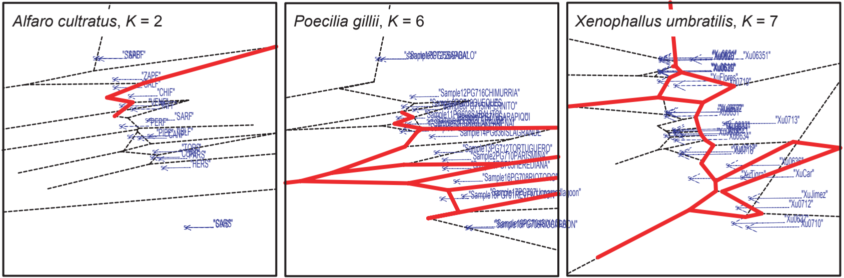

Alfaro cultratus, K = 2
Poecilia gillii, K = 6
Xenophallus umbratilis, K = 7
"ZAPF"
"CHIF"
"SARF"
"PERF"
"HERS"
"Sample12PG716CHIMURRIA"
"Sample14PG636ISLAGRANDE"
"Sample13PG712TORTUGUERO"
"Sample2PG710PARISMINA"
"Xu06351"
"Xu0713"
"XuCar"
"XuJimez"
"Xu0712"
"Xu0710"
